# Genome-wide identification of the *amino acid permease* genes and molecular characterization of their transcriptional responses to various nutrient stresses in allotetraploid rapeseed

**DOI:** 10.1186/s12870-020-02367-7

**Published:** 2020-04-08

**Authors:** Ting Zhou, Cai-peng Yue, Jin-yong Huang, Jia-qian Cui, Ying Liu, Wen-ming Wang, Chuang Tian, Ying-peng Hua

**Affiliations:** 1 School of Agricultural Sciences, Zhengzhou University, Zhengzhou, 450000 China; 2 Sinochem Modern Agricultural Platform, Changchun, 130000 China

**Keywords:** Allotetraploid, Amino acid permease, *Brassica napus*, Genome-wide identification, Nutrient stresses

## Abstract

**Background:**

Nitrogen (N), referred to as a “life element”, is a macronutrient essential for optimal plant growth and yield production. *Amino acid (AA) permease* (*AAP*) genes play pivotal roles in root import, long-distance translocation, remobilization of organic amide-N from source organs to sinks, and other environmental stress responses. However, few systematic analyses of *AAPs* have been reported in *Brassica napus* so far.

**Results:**

In this study, we identified a total of 34 full-length *AAP* genes representing eight subgroups (*AAP1–8*) from the allotetraploid rapeseed genome (A_n_A_n_C_n_C_n_, 2*n* = 4*x* = 38). Great differences in the homolog number among the *BnaAAP* subgroups might indicate their significant differential roles in the growth and development of rapeseed plants. The *BnaAAPs* were phylogenetically divided into three evolutionary clades, and the members in the same subgroups had similar physiochemical characteristics, gene/protein structures, and conserved AA transport motifs. Darwin’s evolutionary analysis suggested that *BnaAAPs* were subjected to strong purifying selection pressure. *Cis*-element analysis showed potential differential transcriptional regulation of *AAPs* between the model Arabidopsis and *B. napus*. Differential expression of *BnaAAPs* under nitrate limitation, ammonium excess, phosphate shortage, boron deficiency, cadmium toxicity, and salt stress conditions indicated their potential involvement in diverse nutrient stress responses.

**Conclusions:**

The genome-wide identification of *BnaAAPs* will provide a comprehensive insight into their family evolution and AAP-mediated AA transport under diverse abiotic stresses. The molecular characterization of core *AAPs* can provide elite gene resources and contribute to the genetic improvement of crop stress resistance through the modulation of AA transport.

## Background

Seed yield and protein content are largely reliant on abundant accumulation of nitrogen (N), which is a macronutrient indispensable for optimal plant growth and organ development [1]. Plants absorb mainly inorganic N nutrients in the form of nitrate (NO_3_^−^) and ammonium (NH_4_^+^), some of which are assimilated into amino acids (AAs) directly in the roots or were translocated to the shoots. In addition, plants can also directly transport AAs and other organic N compounds into roots in soils [2]. The development and metabolism of vegetative and reproductive organs require large numbers of AAs in the phloem. AAs derived from senescent leaves are the major N forms for seed N nutrients [3]. Therefore, efficient uptake and translocation of AAs are favorable for yield production, and also favorable for plant resistance against various stresses [4–7].

More than 100 putative AA transporters (AATs), including the Amino acid–Polyamine–Choline (APC) transporter superfamily and the Usually Multiple Acids Move In and Out Transporters (UmamiT) family, have been characterized in the model Arabidopsis. The APC family can be further grouped into three categories: Amino Acid Permeases (AAPs), Lysine/Histidine-like Transporters (LHTs) and Proline, and Glycine Betaine Transporters (ProTs) [8–10]. Among these AATs, AAPs are considered to be a moderate-affinity system with broad substrate specificity. In plants, AAPs are also involved in various physiological processes, including AA uptake [11], phloem loading or xylem–phloem transfer [12], seed loading [13], and grain yield [14]. AAPs localized on the plasma membrane are involved in cellular H^+^-coupled intake of a broad range of AAs. A recent study has reported that the genetic manipulation of *AAPs* improves AA transport from sources to sinks, which further enhances plant N use efficiency (NUE) [15]. Each member of the *AAP* family shows a specific temporal and spatial expression pattern, which indicates the non-redundant roles of *AAPs* in plants [11].

In Arabidopsis, the AAP family contains eight protein members (AtAAP1–8) that generally transport neutral and acidic AAs with moderate affinity except that AtAAP3 and AtAAP5 also transport basic AAs [16]. *AtAAP1/AtNAT2* (*neutral amino acid transporter 2*) facilitates AA import into the embryo [17, 18]. AtAAP8 contributes to the efficient uptake of AAs into the seed endosperm [19]. *AtAAP3* and *AtAAP5* may be involved in root AA absorption [11, 20]. AtAAP2 and AtAAP6 are reported to function in the xylem-phloem transfer of AAs [21, 22].

The allotetraploid *Brassica napus* (A_n_A_n_C_n_C_n_, 2*n* = 4*x* = 38) is the second most important oleaginous crops worldwide, and it originates from spontaneous hybridization of the diploid *Brassica rapa* (A_r_A_r_, 2*n* = 2*x* = 20) and *Brassica oleracea* (C_o_C_o_, 2*n* = 2*x* = 18) [23–25]. *B. napus* has a relatively higher demand for mineral nutrients, particularly N, than grain crops to achieve optimal seed yield [26]. Indeed, despite its strong capacity of N import, *B. napus* has lower NUE than other major agriculture crops [27], which may be attributed to the fact that senescent leaves easily detach from rapeseed plants before N nutrients, specifically organic amide-N, have been fully remobilized to sink organs [28, 29]. Therefore, improving the N remobilization efficiency in oilseed rape is important for NUE enhancement through molecular modulation of AA transporters, particularly AAPs.

However, few systematic analyses of *AAPs* in *B. napus* have been available so far. In this study, we were aimed to (i) identify the genome-wide *AAP* genes in *B. napus*, (ii) characterize the genomic characteristics and transcriptional responses of the *AAP* gene members to N stresses, including nitrate limitation and ammonium toxicity, and (iii) investigate the transcriptional responses of *AAPs* to other nutrient stresses, including phosphate limitation, boron deficiency, cadmium toxicity, and salt stress. The genome-wide identification and molecular characterization of the *AAP* members indicated their evolutionary conservation and functional divergence between allotetraploid rapeseed and the model Arabidopsis. The global landscapes of the *AAPs* might provide comprehensive insights into the AA import and translocation in allotetraploid rapeseed under diverse nutrient stresses.

## Results

### Genome-wide identification of *AAP* genes

To identify the *AAP* family members in *Brassica* species, we used the AA sequences of AtAAPs to perform BLASTp queries against the genome databases of *B. rapa* (‘Chiifu-401’), *B. oleracea* (‘TO1000’), and *B. napus* (‘Darmor-*bzh*’). The query results revealed great differences in the *AAP* homolog number occurred during the evolutionary process of *Brassica* species (Table 1). As shown in Table 1, *AAPs* had eight members (*AAP1-AAP8*) in the model *A. thaliana*, and each AAP member only had a single copy. A total of 19, 17, and 34 *AAP* homologs were identified in *B. rapa*, *B. oleracea*, and *B. napus*, respectively. It can be found that the number of *AAPs* in *B. napus* was similar to the sum of *AAPs* in both *B. rapa* and *B. oleracea*. Therefore, it could be concluded that most of the *AAPs* were retained during the alloploidy formation of *B. napus*. In *B. napus*, the number of *AAPs* varied from one (*BnaCn.AAP6*) to nine (*BnaAAP8s*) with an average of more than four homologs for each member. The difference in the *AAP* number might suggest their differential expansion patterns of *BnaAAPs* during the allopolyploidy process of *B. napus*.

**Table 1:** Copy number of the *amino acid permease* (*AAP*) genes in Arabidopsis and three *Brassica* species

Item	*Arabidopsis thaliana* (125 Mb)	*Brassica rapa* (465 Mb)	*Brassica oleracea* (485 Mb)	*Brassica napus* (1130 Mb)
*AAP1*	1	3	2	6
*AAP2*	1	2	2	4
*AAP3*	1	1	1	3
*AAP4*	1	2	2	4
*AAP5*	1	3	3	5
*AAP6*	1	1	1	1
*AAP7*	1	1	1	2
*AAP8*	1	6	5	9
Total	8	19	17	34

### Genomic distribution and gene expansion of *BnaAAPs*

**Fig. 1:**
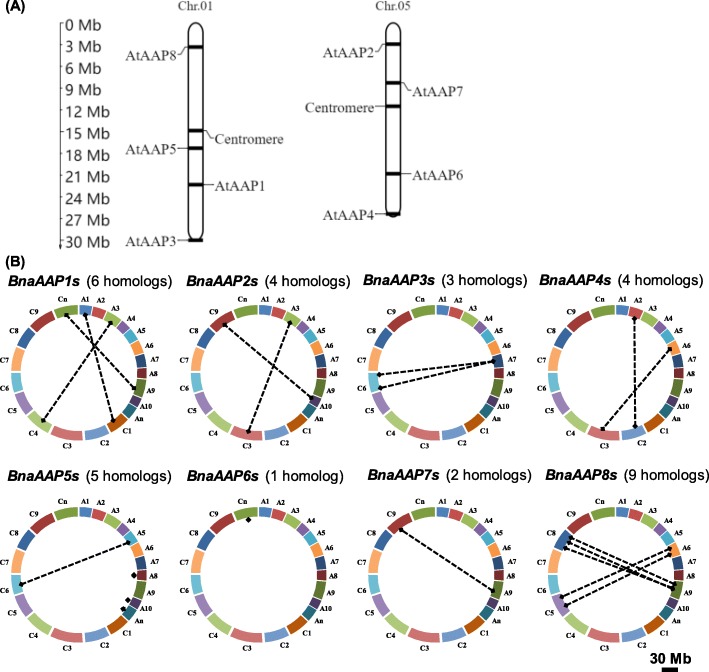
Physical mapping and syntenic analysis of the *amino acid permease* (*AAP*) family genes in *Arabidopsis thaliana* and *Brassica napus*. **a** Genomic position of the Arabidopsis *AAP* genes (**a**) and their homologs in *B. napus* (**b**). The *BnaAAP* homologs between the An and Cn sub-genomes of rapeseed are connected by crashed lines. The length scale of *B. napus* chromosomes (An subgenome: A1-A10; Cn subgenome: C1-C9) is 30.0 Mb

Through physical position identification, we found that eight *AAPs* in Arabidopsis showed strong preference for chromosomal distribution: four of them are located on the Chr. 01 (*AtAAP1*, *AtAAP3*, *AtAAP5* and *AtAAP8*) and the other four on Chr. 05 (*AtAAP2*, *AtAAP4*, *AtAAP6* and *AtAAP7*) (Table 2, Fig. 1a). To further explore the genomic distribution and gene expansion of *BnaAAPs*, we retrieved the DNA sequences of *BnaAAPs* in the *Brassica* and CNS-Genoscope databases. Different from the intensive distribution of *AtAAPs*, the *BnaAAPs* were physically scattered on 12 chromosomes (A subgenome: A1, A2, A3, A6, A7, A9, and A10; C subgenome: C1, C2, C3, C6, and C9) (Table 2). In the A subgenome of *B. napus*, Chromosome A9 exhibits the largest chromosome size and the second largest gene number according to the rapeseed genome annotation (http://www.genoscope.cns.fr/brassicanapus/). Our study showed that Chromosome A9 has the largest *BnaAAP* gene number, i.e. four, including *BnaA9.AAP1* (BnaA09g14700D), *BnaA9.AAP7* (BnaA09g05130D), *BnaA9.AAP8a* (BnaA09g57230D), and *BnaA9.AAP8b* (BnaA09g57240D) (Table 2). In the C subgenome, more *AAPs* were identified on both Chromosome C6 and C8 than on the other seven chromosomes (C1-C5, C7, and C9). The homolog number of eight *BnaAAP* family genes varies from one to three, and they are relatively evenly distributed on different chromosomes (Table 2).

**Table 2:** Molecular characterization of the *amino acid permease* (*AAP*) genes in *Arabidopsis thaliana* and *Brassica napus*

Gene ID	Gene name	Block	CDS (bp)	Exon/ intron	Amino acid (aa)	Ka	Ks	Ka/Ks	Divergent time (Mya)
At1g58360	*AtAAP1*	D	1458	6/5	485				
BnaA01g21750D	*BnaA1.AAP1*	D	1524	6/5	507	0.0394	0.3931	0.1002	13.10
BnaA03g59400D	*BnaA3.AAP1*	D	1623	8/7	540	0.0565	0.3987	0.1417	13.29
BnaA09g14700D	*BnaA9.AAP1*	D	1440	6/5	479	0.0466	0.4043	0.1153	13.48
BnaC01g42990D	*BnaC1.AAP1*	D	1524	6/5	507	0.0405	0.3747	0.1081	12.49
BnaC04g18440D	*BnaC4.AAP1*	D	1455	6/5	484	0.0489	0.3854	0.1269	12.85
BnaCnng25620D	*BnaCn.AAP1*	D	1440	6/5	479	0.0489	0.3965	0.1233	13.22
At5g09220	*AtAAP2*	R	1482	6/5	493				
BnaA03g02650D	*BnaA3.AAP2*	R	1464	7/6	487	0.0293	0.4707	0.0622	15.69
BnaA10g22670D	*BnaA10.AAP2*	R	1458	6/5	485	0.0366	0.5027	0.0728	16.76
BnaC03g03750D	*BnaC3.AAP2*	R	1464	6/5	487	0.0313	0.4676	0.0669	15.59
BnaC09g47230D	*BnaC9.AAP2*	R	1458	6/5	485	0.0437	0.4787	0.0913	15.96
At1g77380	*AtAAP3*	E	1431	7/6	476				
BnaA07g33510D	*BnaA7.AAP3*	E	1431	7/6	476	0.0374	0.3945	0.0948	13.15
BnaC06g38080D	*BnaC6.AAP3a*	E	1431	7/6	476	0.0386	0.3865	0.0999	12.88
BnaC06g38090D	*BnaC6.AAP3b*	E	1431	7/6	476	0.0408	0.3847	0.1061	12.82
At5g63850	*AtAAP4*	X	1401	6/5	466				
BnaA02g33930D	*BnaA2.AAP4*	X	1401	6/5	466	0.0304	0.5479	0.0555	18.26
BnaA06g22970D	*BnaA6.AAP4*	X	1401	5/4	466	0.0392	0.4567	0.0858	15.22
BnaC02g42740D	*BnaC2.AAP4*	X	1401	5/4	466	0.0306	0.5398	0.0567	17.99
BnaC03g50500D	*BnaC3.AAP4*	X	1401	5/4	466	0.0423	0.4367	0.0969	14.56
At1g44100	*AtAAP5*	C	1443	5/4	480				
BnaAnng17090D	*BnaAn.AAP5*	C	1248	4/3	415	0.0804	0.4964	0.1620	16.55
BnaA08g04440D	*BnaA8.AAP5*	C	1446	5/4	481	0.0840	0.5449	0.1542	18.16
BnaA05g18660D	*BnaA5.AAP5*	C	1464	5/4	487	0.0801	0.5204	0.1539	17.35
BnaA10g08840D	*BnaA10.AAP5*	C	1431	5/4	476	0.1206	0.5015	0.2405	16.72
BnaC06g00580D	*BnaC6.AAP5*	C	1431	5/4	476	0.1149	0.4909	0.2341	16.36
At5g49630	*AtAAP6*	W	1446	6/5	481				
BnaCnng14480D	*BnaCn.AAP6*	W	1440	6/5	479	0.0560	0.4756	0.1177	15.85
At5g23810	*AtAAP7*	Q	1404	6/5	467				
BnaA09g05130D	*BnaA9.AAP7*	Q	1413	7/6	470	0.1092	0.3543	0.3082	11.81
BnaC09g04700D	*BnaC9.AAP7*	Q	1059	6/5	352	0.1169	0.3813	0.3066	12.71
At1g10010	*AtAAP8*	A	1428	6/5	475				
BnaA06g38000D	*BnaA6.AAP8a*	A	1401	6/5	466	0.1387	0.4977	0.2787	16.59
BnaA06g38010D	*BnaA6.AAP8b*	A	1389	6/5	462	0.1385	0.5880	0.2355	19.60
BnaA09g57230D	*BnaA9.AAP8a*	A	1446	6/5	481	0.1387	0.4977	0.2787	16.59
BnaA09g57240D	*BnaA9.AAP8b*	A	1584	6/5	527	0.1385	0.5880	0.2355	19.60
BnaC05g49200D	*BnaC5.AAP8a*	A	1410	7/6	469	0.1361	0.5426	0.2508	18.09
BnaC05g49210D	*BnaC5.AAP8b*	A	1449	6/5	482	0.1385	0.5880	0.2355	19.60
BnaC08g42410D	*BnaC8.AAP8a*	A	1446	6/5	481	0.1508	0.2216	0.6805	7.39
BnaC08g42420D	*BnaC8.AAP8b*	A	1515	7/6	504	0.1463	0.5326	0.2747	17.75
BnaC08g42430D	*BnaC8.AAP8c*	A	1446	6/5	481	0.1321	0.5257	0.2513	17.52

Note: *CDS* coding sequence, *Ka* non-synonymous nucleotide substitution rate, *Ks* synonymous nucleotide substitution rate

**Fig. 2:**
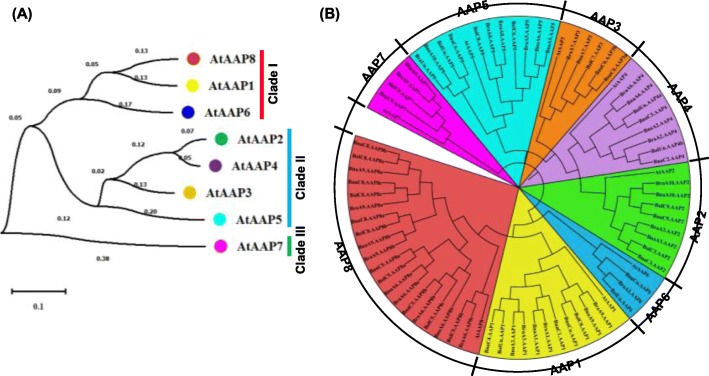
Phylogeny analysis of the *amino acid permease* (*AAP)* genes in *Arabidopsis thaliana* and *Brassica* crops. **a-b** Phylogeny analysis of *AtAAPs* (**a**) and the *AAPs* in *Brassica* species (**b**), including *A. thaliana*, *B. rapa*, *B. oleracea*, and *B. napus*. The AAP protein sequences were multi-aligned using the ClustalW program, and then an unrooted phylogenetic tree was constructed using MEGA 6.06 with the neighbor-joining method. The percentages of replicate trees, in which the associated taxa clustered together in the bootstrap test (1000 replicates), are shown next to the branches. The tree is drawn to scale, with branch lengths in the same units as those of the evolutionary distances used to infer the phylogenetic tree. The evolutionary distances were computed with the Poisson correction method, and are in the units of the number of amino acid substitutions per site

Gene family expands mainly via three pathways: tandem duplication, segmental duplication, and whole-genome duplication [30]. The *B. napus* progenitor diploids (*B. rapa* and *B. oleracea*) are ancient polyploids, and large-scale chromosomal rearrangements have occurred since their evolution from *Amborella trichopoda*, a low chromosome number progenitor [31]. Comparative genomics reveals that the Arabidopsis genome is divided into 24 ancestral crucifer blocks, which are labeled A-X [32]. Table 2 shows that the *AtAAP* genes and their corresponding homologs in *B. napus* are located in the same chromosomal blocks. To further understand the expansion patterns of *BnaAAPs*, we investigated their duplication events. The results showed that the *AAP* family members in *B. napus* were derived from the corresponding ancestors in *B. rapa* or *B. oleracea*, except *BnaAAP8s*, which expanded potentially through tandem duplication (Figs. 1b and 2). Based on the above result, we presumed that segmental duplication might be a main contributor to the *BnaAAP* family expansion.

### Phylogeny analysis of BnaAAPs

To elucidate the molecular evolution and phylogenetic relationships among the AAP proteins, we constructed two unrooted phylogenetic trees involving AtAAPs and their homologs in *Brassica* species (Fig. 2). In Arabidopsis, according to the phylogeny relationships, we made the first attempt to classify the AAP family members into three major clades: Clade I (AtAAP1, AtAAP6, and AtAAP8), Clade II (AtAAP2, AtAAP3, AtAAP4, and AtAAP5), and Clade III (AtAAP7) (Fig. 2a). Further, we performed a phylogeny analysis of 78 AAP proteins in *A. thaliana* and three *Brassica* crop species, including *B. rapa*, *B. oleracea*, and *B. napus*. The phylogenetic tree was also subclassified into eight smallest categories, and the AAP members from different species were closely clustered with their corresponding homologs in *A. thaliana* (Fig. 2b). What is noteworthy, the *AAP* genes of *B. napus* were clustered into the smallest clades with their corresponding homologs in the diploid ancestor *B. rapa* or *B. oleracea* (Additional file: Figure S1). The result indicated that the AAP proteins divergence was prior to the *Brassica* speciation. We also observed that most of the AAP proteins within each subfamily had very short branch lengths (Fig. 2b), suggesting a recent genetic divergence.

### Molecular characterization of BnaAAPs

**Fig. 3:**
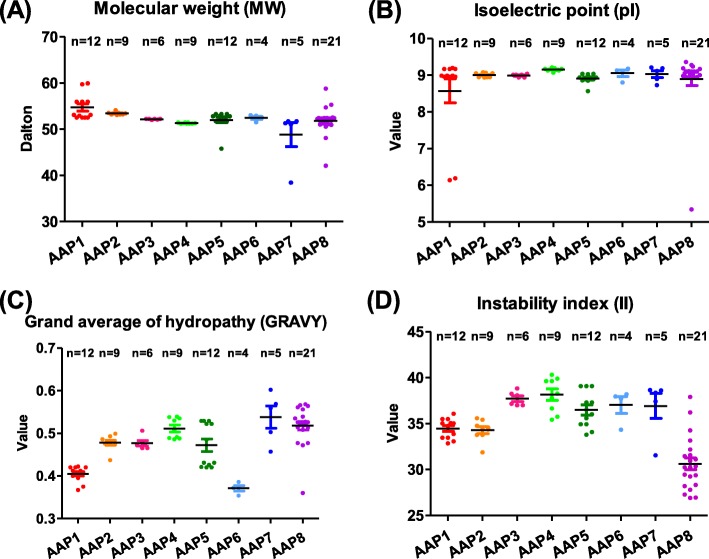
Molecular characterization of the amino acid permease (AAP) proteins in *Brassica napus*. **a-d** Molecular weights (MWs, **a**), theoretical isoelectric points (pIs, **b**), grand average of hydropathy (GRAVY, **c**) values and instability indices (IIs, **d**) of the BnaAAP proteins. The GRAVY value is defined as the sum of hydropathy values of the amino acids divided by the protein length. An II value < 40.0 indicates that the protein is stable

To understand the molecular characteristics of the BnaAAP proteins, we calculated the physicochemical parameters of each BnaAAP protein using ExPASy. The results showed that most proteins in the same AAP subfamily had similar parameters (Table 2). In total, the coding sequence (CDS) lengths of *BnaAAPs* varied from 1059 bp (*BnaC9.AAP7*) to 1584 bp (*BnaA9.AAP8b*), corresponding to the variation of the deduced AA number from 352 to 527 (Table 2). Most of the computed molecular weights (MWs) of the BnaAAP proteins ranged from 50.0 kD to 60.0 kD, except that those of BnaA10.AAP5 (45.8 kD) and BnaC9.AAP7 (38.4 kD) were smaller than 50.0 kD (Fig. 3a, Additional file 1: Table S1). The theoretical isoelectric points (pIs) of BnaAAPs varied from 6.19 (BnaA3.AAP1) to 9.28 (BnaA6.AAP8b), with most > 7.0 except that of BnaA3.AAP1 (6.19) (Fig. 3b, Additional file 1: Table S1). The grand average of hydropathy (GRAVY) value is calculated as the sum of hydropathy values of the AAs divided by the protein length. The results showed that the GRAVY values of the BnaAAP members ranged from 0.360 (BnaC8.AAP8a) to 0.566 (BnaC5.AAP8b) (Fig. 3c, Additional file 1: Table S1). Therefore, all the BnaAPPs were presumed to be hydrophobic. The instability indices (IIs) values of all the BnaAAPs were < 40.0 (Fig. 3d, Additional file 1: Table S1), which showed strong protein stability. The online WoLF PSORT was used to predict the subcellular localization of eight AtAAPs and 34 BnaAAPs. The result indicated that they were localized in the plasma membrane, suggesting that they might be responsible for the trans-membrane transport of AAs. We used the TMHMM tool to characterize the transmembrane structures of AAPs in *A. thaliana* and *B. napus*, and found that AtAAPs and BnaAAPs had nine to ten membrane-spanning regions (Additional file 1: Figure S2). In detail, the AtAAP5/BnaAAP5s, AtAAP6/BnaAAP6s, and AtAAP7/BnaAtAAP7s had ten transmembrane regions, and the other five subgroup members had nine membrane-spanning regions Additional file 1: Figure S2). Similarly to the AtAAPs without signal peptides (Additional file 1: Figure S3), the BnaAPPs were observed to have no signal peptides, either. The Recombinant Protein Solubility Prediction (version 2009) indiated that the recombinant BnaAAPs would be insoluble when these proteins were overexpressed in *E. coli*.

### Identification of evolutionary selection pressure on *BnaAAPs*

To characterize selection pressure on the *BnaAAPs* during the evolutionary process, we used the orthologous *AAP* gene pairs between *B. napus* and *A. thaliana* to determine the values of synonymous (Ks) and non-synonymous (Ka) nucleotide substitution rates, and Ka/Ks (Table 2). The Ka values of *BnaAAPs* ranged from 0.0293 (*BnaA3.AAP2*) to 0.1508 (*BnaC8.AAP8a*) with an average of 0.05, and the Ks values of *BnaAAPs* ranged from 0.2216 (*BnaA3.AAP2*) to 0.5880 (*BnaC8.AAP8a*) with an average of 0.5199. Further, we found that all the Ka/Ks values of *BnaAAPs* were < 1.0 (Table 2). Therefore, we presumed that the *BnaAAPs* might have experienced a very strong negative selection to preserve their function.

The Ks values of the duplicated homologs among gene families are usually presumed to be molecular clocks, and these Ks values are assumed to be constant over time. It has been reported that the segregation between the model Arabidopsis and its-derived *Brassica* plants occurred 12–20 million years ago (Mya) [33]. In this study, our results showed that most of *BnaAAPs* might diverge from *AtAAPs* approximately 12.0–18.0 Mya except *BnaC8.AAP8a*, implying that the *Brassica* speciation was accompanied by the divergence of the *AAP* genes.

### Conserved domain, exon-intron organization, gene interaction, and transcriptional regulatory analysis of *BnaAAPs*

**Fig. 4:**
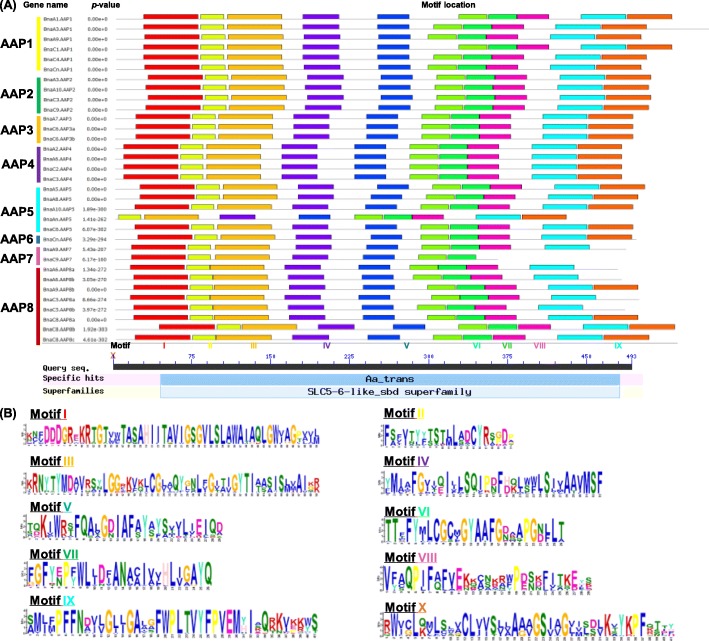
Identification and characterization of the conserved motifs in the amino acid permease (AAP) proteins in *Arabidopsis thaliana* and *Brassica napus*. Molecular identification (**a**) and sequence characterization (**b**) of the conserved motifs in the AAP proteins in *A. thaliana* and *B. napus*. In **a**, the boxes with different colors indicate different conserved motifs (motif 1–15), and grey lines represent the AAP protein regions without detected motifs. Aa_trans indicates the amino acid transport regions; SCL5–6-like_sbd refers to the solute carrier (SCL) families 5 and 6-like proteins with solute binding domain (sbd). In **b**, the larger the fonts, the more conserved the motifs

AA residues are thought to be functionally or structurally significant if they are evolutionarily conserved. The output of MEME showed that the eight BnaAAP subfamilies had conserved domains (Fig. 4a). Further, we identified that these conserved domains belong to the amino acid transporter family, which is part of the solute carrier (SCL) superfamily proteins with solute binding domains (Fig. 4a). Among the ten conserved domains we defined, the AA sequences of Motif I and IX exhibited the highest identity (Fig. 4b), thereby which could be used as the identification indicators of the AAP family members.

**Fig. 5:**
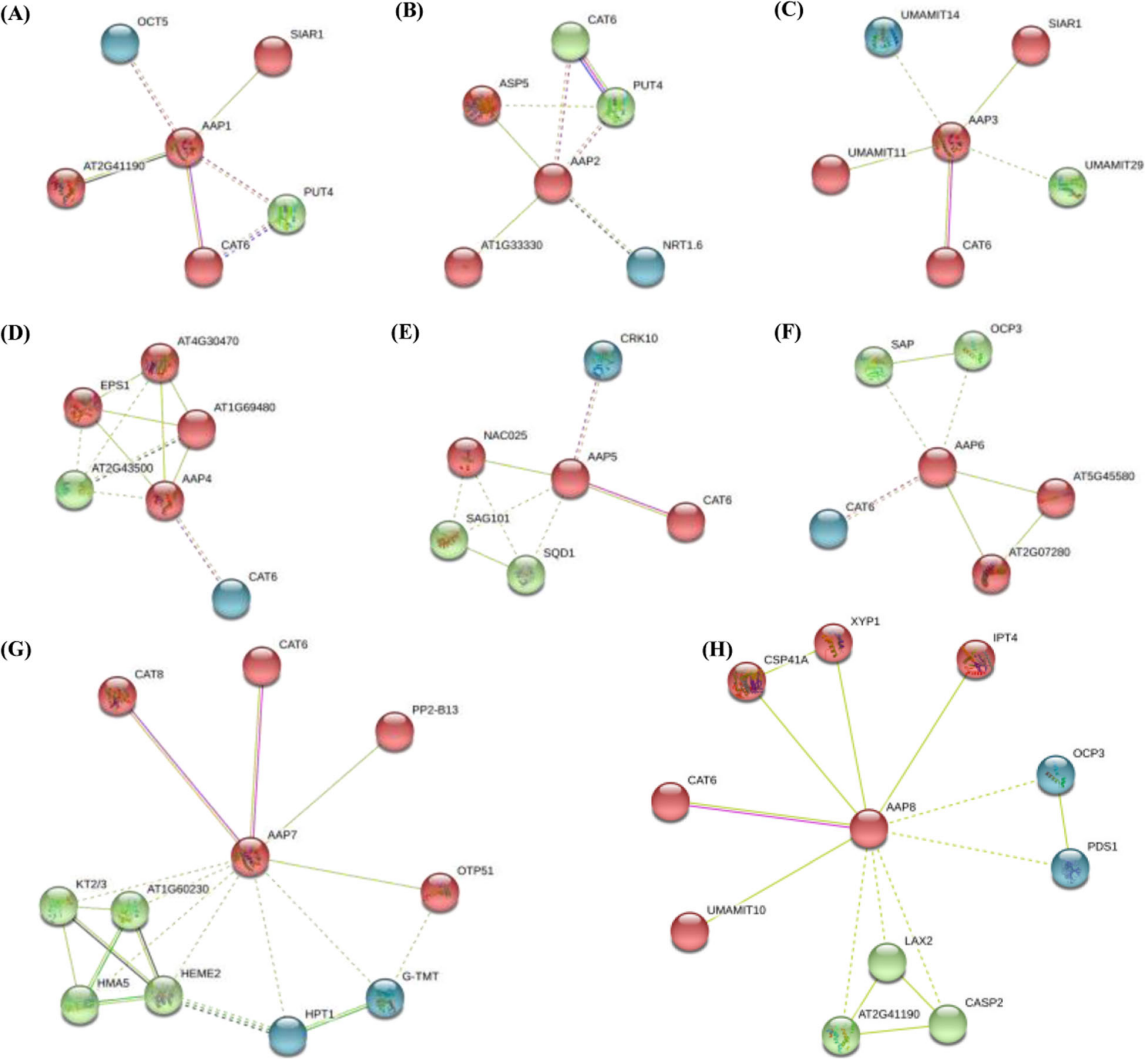
Protein-protein interaction networks of the amino acid permease (AAP) proteins in *Brassica napus*. The interaction networks of the AAP1 (**a**), AAP2 (**b**), AAP3 (**c**) AAP4 (**d**), AAP5 (**e**), AAP6 (**f**), AAP7 (**g**) and AAP8 (**h**) and other proteins were constructed by the STRING web-server. Network nodes represent proteins. The network is clustered into three clusters, which are represented with red, green, and blue nodes, respectively. Colored nodes: query proteins and first shell of protein interactors; white nodes: the second shell of protein interactors. Empty nodes: proteins of unknown 3D structure; filled nodes: some 3D structure is known or predicted. Edges represent protein-protein associations

To further identify the protein(s) potentially interacting with the AAP family members, we constructed a protein interaction network involving direct (physical) and indirect (function) association by using the STRING database based on either known experimental or predicted interactions. As shown in Fig. 5, all the AAP proteins consistently interacted with CAT6, a cation AA transporter (CAT) belonging to the AA polyamine choline (APC) family [34]. Besides, some other AA transporters, such as CAT8, SIAR1 (Silique Are Red 1), and the UMAMIT (Usually Multiple Acids Move In and out Transporters) family members, were also observed to interact with the AAP proteins (Fig. 5).

**Fig. 6:**
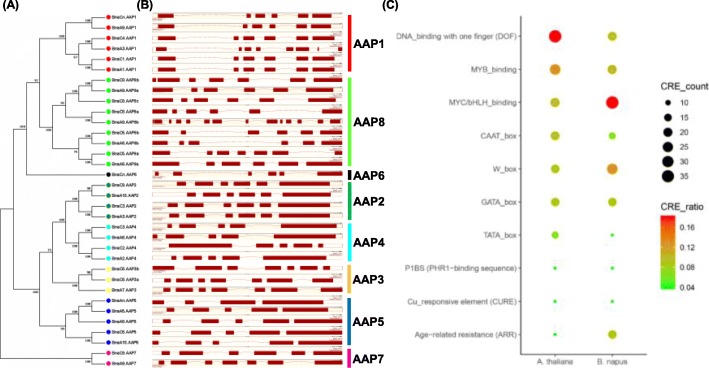
Phylogeny relationship (**a**), exon-intron organization **b**) of the *amino acid permease* (*AAP*) genes and enrichment analysis (**c**) of the *cis*-acting regulatory elements (CREs) in the *AAP* promoter regions in *Brassica napus*. For each *AAP*, a 2.0-kb genomic sequence in the upstream from the start codon (ATG) was downloaded from the TAIR (https://www.arabidopsis.org/) website and *B. napus* Genome Browser (http://www.genoscope.cns.fr/brassicanapus/) [24]. Subsequently, we submitted these sequences to the PLACE v. 30.0 (http://www.dna.affrc.go.jp/PLACE/) program [35] to identify putative CREs and calculate the CRE number along the 2.0 kb gene promoter region. In the scatter plot of **c**, the larger the circle sizes, the more the corresponding CREs

The exon-intron number and organization are indicative of evolutionary imprints within gene families. Considering this, we identified the gene structures of *BnaAAPs* by comparing the genomic DNA sequences with their corresponding CDSs. As shown in Fig. 6(a, b) and Table 2, in general, most *AtAAP*s had similar gene structures to their homologs in *B. napus*, which indicated their conserved functionality between the ancestor Arabidopsis and *B. napus*. However, we also observed some structure variations within a *BnaAAP* subgroup (Table 2). The exon/intron number variations, potentially caused by alternative splicing, might contribute to the functional differentiation of different *BnaAAP* members.

Transcription factors (TFs), binding to *cis*-acting regulatory elements (CREs) in the promoters of their target genes, play important roles in the transcriptional regulation [36]. To identify the core TFs regulating *BnaAAPs*, the 2.0-kb upstream sequences of the *BnaAAP* start condons were used to explore the over-accumulated CREs (Fig. 6). The results showed that all the *BnaAAP* family genes had various types of CREs in their promoter regions, which suggested that the complicated regulatory networks of *BnaAAPs* might be involved in their transcriptional regulation. Apart from the common CREs, such as the TATA box and the CAAT box, we also identified that three terms, namely, GATA-box, W-box (TGAC), and MYC/bHLH-binding elements were most highly enriched in the *BnaAAP* promoter regions (Fig. 6c). In both Arabidopsis and *B. napus*, the DNA-binding with one finger (Dof), MYB-binding, and MYC/bHLH-binding elements were highly enriched (Fig. 6c). These results showed that on one hand, there Arabidopsis and *B. napus* shared the common regulatory mechanisms of *AAPs*, on the other hand, these two species had their own specific regulatory mechanisms.

### Transcriptional analysis of *BnaAAP*s under diverse nutrient stresses

To identify the roles of *BnaAAPs* in regulating rapeseed against various nutrient stresses, we examined their transcriptional responses under these circumstances. First, we investigated the transcriptional patterns of *AtAAPs* in various tissues through the TAIR eFP Browser. The results showed that the *AtAAP1*, *AtAAP2*, *AtAAP4*, *AtAAP5*, and *AtAAP6* genes were highly expressed in cauline and senescent leaves (Additional file 1: Figure S4A, B, D, E, and F), which indicated that these genes might play pivotal roles in the translocation of AAs from source leaves to sink organs. Furthermore, both *AtAAP*3 and *AtAAP8* had the highest expression levels in the embryo seeds (Additional file 1: Figure S4C and H), suggesting that they might be involved in seed development. The preferential expression of *AtAAP7* in the second internodes (Additional file 1: Figure S3G) suggested its participation in long-distance translocation of amide-N nutrients. The differential expression of *AAPs* implied their specific roles in plant growth and development.

**Fig. 7:**
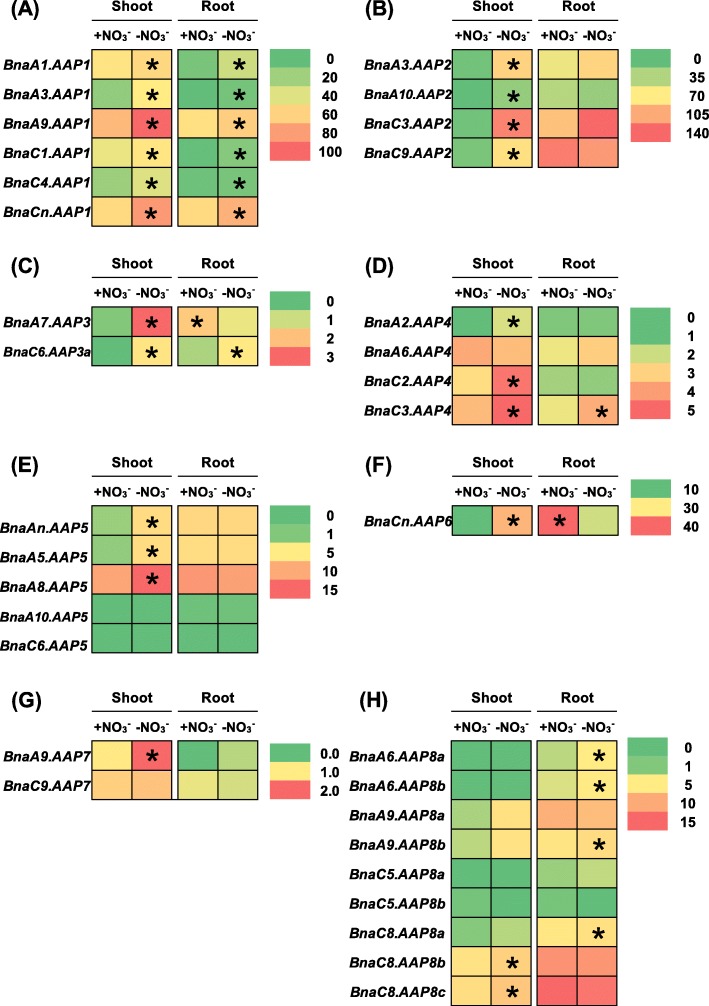
The qRT-PCR-assisted transcriptional characterization of the *amino acid permease* (*AAP*) genes in *Brassica napus* under different nitrate (NO_3_^−^) supply levels. Differential expression of the *BnaAAP1* (**a**), *BnaAAP2*(**b**), *BnaAAP3* (**c**), *BnaAAP4* (**d**), *BnaAAP5*(**e**), *BnaAAP6* (**f**), *BnaAAP7* (**g**) and *BnaAAP8* (**h**) genes under high and low nitrate conditions. For the transcriptional analysis, the 7-d-old uniform *B. napus* seedlings after seed germination were cultivated under high (+, 6.0 mM) nitrate for 10 d, and then were transferred to low (−, 0.30 mM) nitrate for 3 d until sampling. The shoots and roots were individually sampled, and each sample includes three independent biological replicates. The significance level of *P* < 0.05 is used as the threshold to identify the differential expression of *BnaAAPs* under high and low nitrate treatments. The differentially expressed genes with higher expression between different treatments in the shoots or roots are indicated with asterisks

**Fig. 8:**
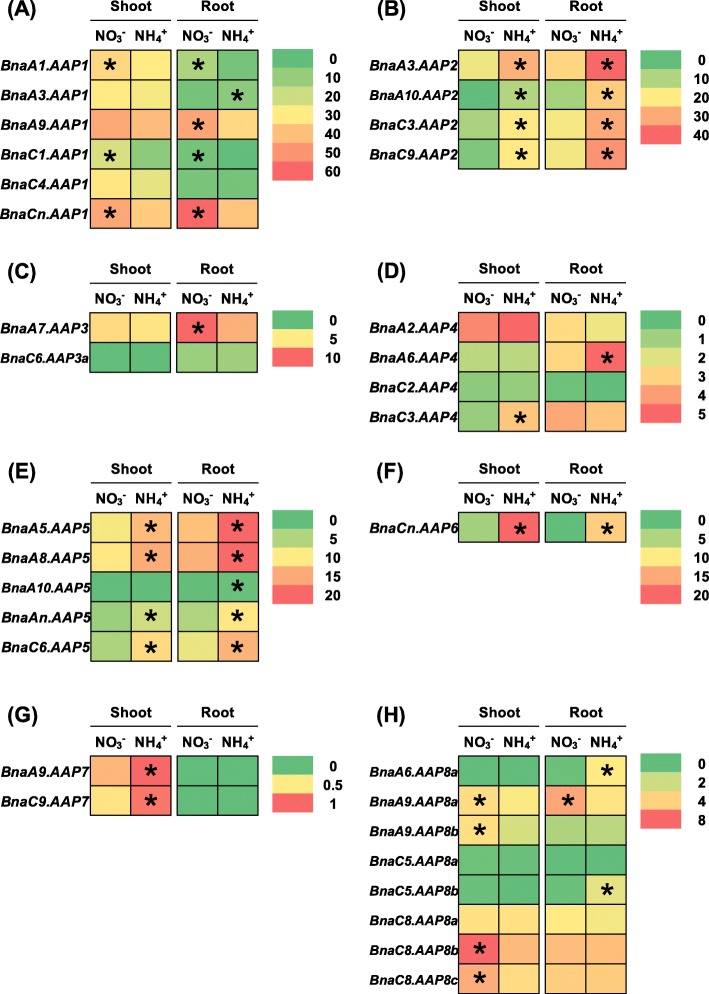
The qRT-PCR-assisted transcriptional characterization of the *amino acid permease* (*AAP*) genes in *Brassica napus* under different nitrogen (N) form conditions. Differential expression of the *BnaAAP1* (**a**), *BnaAAP2* (**b**), *BnaAAP3* (**c**), *BnaAAP4* (**d**), *BnaAAP5* (**e**), *BnaAAP6* (**f**), *BnaAAP7* (**g**) and *BnaAAP8* (**h**) genes under nitrate (NO_3_^−^) and ammonium (NH_4_^+^) conditions. For the transcriptional analysis, the rapeseed seedlings were first hydroponically cultivated under 6.0 mM nitrate (NO_3_^−^) for 10 d, and then were transferred to an N-free solution for 3 d. Subsequently, the above seedlings were sampled after treatment with 6.0 mM ammonium (NH_4_^+^) for 3 d. The shoots and roots were individually sampled, and each sample includes three independent biological replicates. The significance level of *P* < 0.05 is used as the threshold to identify the differential *BnaAAP* expression under high and low nitrate treatments. The differentially expressed genes with higher expression between different treatments in the shoots or roots are indicated with asterisks

The above-mentioned results indicated that multiple copies of each *AAP* homolog occurred in allotetraploid rapeseed (Fig. 1), and that transcriptional identification of the core *AAP* members were very important for the in-depth understanding of the *BnaAAP* function. Rapeseed has a high demand for N nutrients whereas it shows very low NUE [26, 27]. Under N stresses, the metabolic profiles of AAs were significantly altered in plants [37]. Under nitrate limitation condition, 26 members of 34 *BnaAAPs* were differentially expressed in rapeseed plants compared with the condition of sufficient nitrate supply (Fig. 7, Additional file 1: Tables S2, S3). In detail, most of the differentially expressed genes (DEGs) were upregulated in the shoots or roots under nitrate deficiency except that *BnaA7.AAP3* and *BnaCn.AAP6* were downregulated in the roots. It should be noted that among the eight *BnaAAP* subfamilies, the expression of all the *BnaAAP1s* was consistently induced in both the shoots and roots (Fig. 7a). Under ammonium toxicity, we identified a total of 26 *BnaAAP* DEGs in the shoots and roots relative to the condition of nitrate sufficiency (Fig. 8). In the shoots, we found that the DEGs of only *BnaAAP1s* and *BnaAAP8s* showed higher expression levels under nitrate sufficiency than under ammonium toxicity (Fig. 8a, h), and the DEGs of other *BnaAAPs* were upregulated only when ammonium was supplied as the sole N nutrient source (Fig. 8b-g). In the roots, the expression of most family members (*BnaAAP2s*, *BnaAAP4s*, *BnaAAP5s*, *BnaAAP6s*, and *BnaAAP8s*) was induced under ammonium toxicity (Fig. 8b, d, e, f, h) whereas the differential expression profiling of *BnaAAP1s* (except *BnaA3.AAP1*) and *BnaAAP3s* presented the opposite pattern (Fig. 8a, c).

**Fig. 9:**
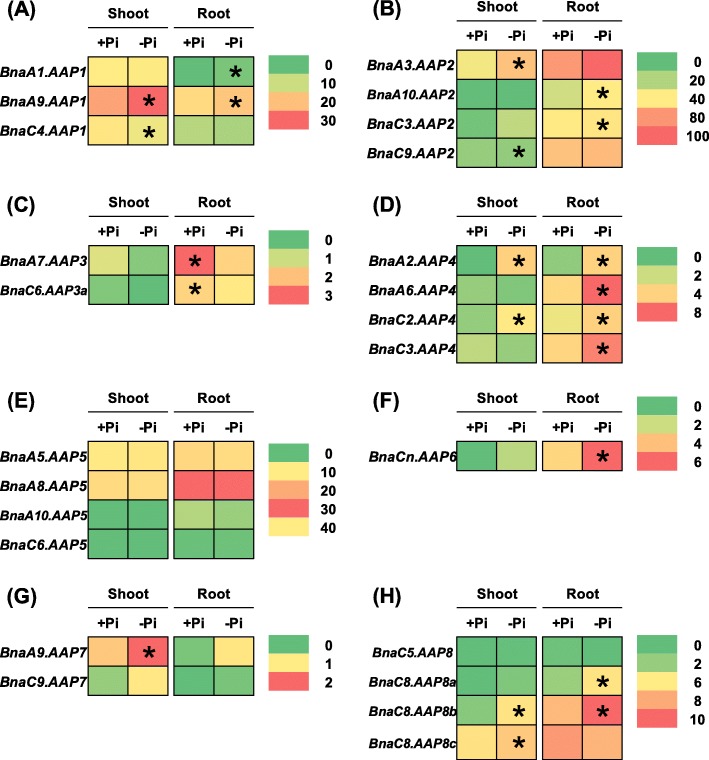
The qRT-PCR-assisted transcriptional characterization of the *amino acid permease* (*AAP*) genes in *Brassica napus* under different phosphate (Pi) levels. Differential expression of the *BnaAAP1* (**a**), *BnaAAP2* (**b**), *BnaAAP3* (**c**), *BnaAAP4* (**d**), *BnaAAP5* (**e**), *BnaAAP6* (**f**), *BnaAAP7* (**g**) and *BnaAAP8* (**h**) genes under high (+, 250 μM) phosphate and low (−, 5 μM) phosphate levels. For the transcriptional analysis, the 7-d-old uniform *B. napus* seedlings after seed germination were first hydroponically grown under 250 μM phosphate (KH_2_PO_4_) for 10 d, and then were transferred to 5 μM phosphate for 3 d until sampling. The shoots and roots were individually sampled, and each sample includes three independent biological replicates. The significance level of *P* < 0.05 is used as the threshold to identify the differential *BnaAAP* expression under high and low phosphate treatments. The differentially expressed genes with higher expression between different treatments in the shoots or roots are indicated with asterisks

Phosphorus is one of the mineral nutrients required for plant growth, and it is widely used as fertilizers in agricultural production. Hu et al. [38] found that phosphate availability modulates the expression of nitrate-responsive genes, and that the NRT1.1B-SPX4 module is possibly involved in phosphate-regulated nitrate response, which indicates the interaction between N and phosphorus nutrients. Under phosphate limitation condition, a total of 15 *BnaAAP* DEGs were identified in the shoots or roots (Fig. 9, Additional file 1: Table S2). In the shoots, no differential expression of *BnaAAP5s* and *BnaAAP6s* was observed between sufficient phosphate and insufficient phosphate conditions (Fig. 9e, f). However, the DEGs of other *BnaAAPs* were upregulated by limited phosphate (Fig. 9a-d, g-h). In the roots, 13 *BnaAAPs* exhibited differential expression, however, we did not identify the differential expression of *BnaAAP5s* and *BnaAAP7s* between sufficient phosphate and insufficient phosphate conditions (Fig. 9e, g). The DEGs of *BnaAAP1s*, *BnaAAP2s*, *BnaAAP4s*, *BnaAAP6s*, and *BnaAAP8s* presented higher expression levels under phosphate insufficiency than under phosphate sufficiency condition (Fig. 9a, b, d, f, h). By contrast, insufficient phosphate supply repressed the expression of *BnaAAP3s* in the roots (Fig. 9c).

**Fig. 10:**
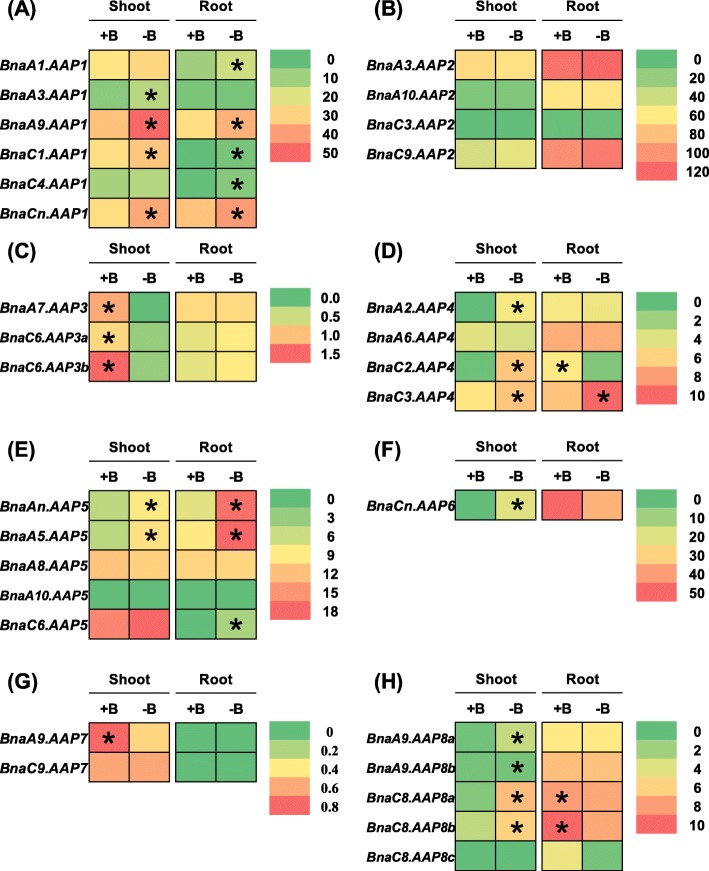
The qRT-PCR-assisted transcriptional characterization of the *amino acid permease* (*AAP*) genes in *Brassica napus* under different boron (B) levels. Differential expression of the *BnaAAP1* (**a**), *BnaAAP2* (**b**), *BnaAAP3* (**c**), *BnaAAP4* (**d**), *BnaAAP5* (**e**), *BnaAAP6* (**f**), *BnaAAP7* (**g**) and *BnaAAP8* (**g**) genes under high (+, 25 μM) and low (−, 0.25 μM) boron supply levels. For the transcriptional analysis, the 7-d-old uniform *B. napus* seedlings after seed germination were first hydroponically grown under 10 μM H_3_BO_3_ for 10 d, and then were transferred to 0.25 μM H_3_BO_3_ for 3 d until sampling. The shoots and roots were individually sampled, and each sample includes three independent biological replicates. The significance level of *P* < 0.05 is used as the threshold to identify the differential *BnaAAP* expression under high and low boron treatments. The differentially expressed genes with higher expression between different treatments in the shoots or roots are indicated with asterisks

Boron is an essential micronutrient for vegetative and reproductive growth in plants [39], and our previous studies reveal that *B. napus* is hypersensitive to boron deficiency [40, 41]. In rapeseed plants, Wang et al. [42] found that boron deprivation enhances protein degradation and influences AA metabolism. Gas chromatography-mass spectrometry (GC-MS)-based metabolomics reveals that AA accumulation is increased in boron-deficient citrus [43]. Under deficient boron condition, we identified a total of 21 *BnaAAP* DEGs in the shoots and roots (Fig. 10a, c-h, Additional file 1: Table S2) whereas we did not detect any differential expression of *BnaAAP2s* (Fig. 10b, Additional file 1: Table S2). In the shoots, the expression of the *BnaAAP1*, *BnaAAP4*, *BnaAAP5*, *BnaAAP6*, and *BnaAAP8* DEGs was significantly upregulated by boron deficiency whereas the *BnaAAP3* and *BnaAAP7* DEGs showed the opposite expression patterns (Fig. 10c, g; Additional file 1: Table S2). In the roots, we found that the low boron significantly induced the expression of the *BnaAAP1*, *BnaC3.AAP4*, and *BnaAAP5* DEGs (Fig. 10a, e; Additional file 1: Table S2), whereas repressed the expression of the *BnaC2.AAP4* and *BnaAAP8* DEGs (Fig. 10d, h; Additional file 1: Table S2).

**Fig. 11:**
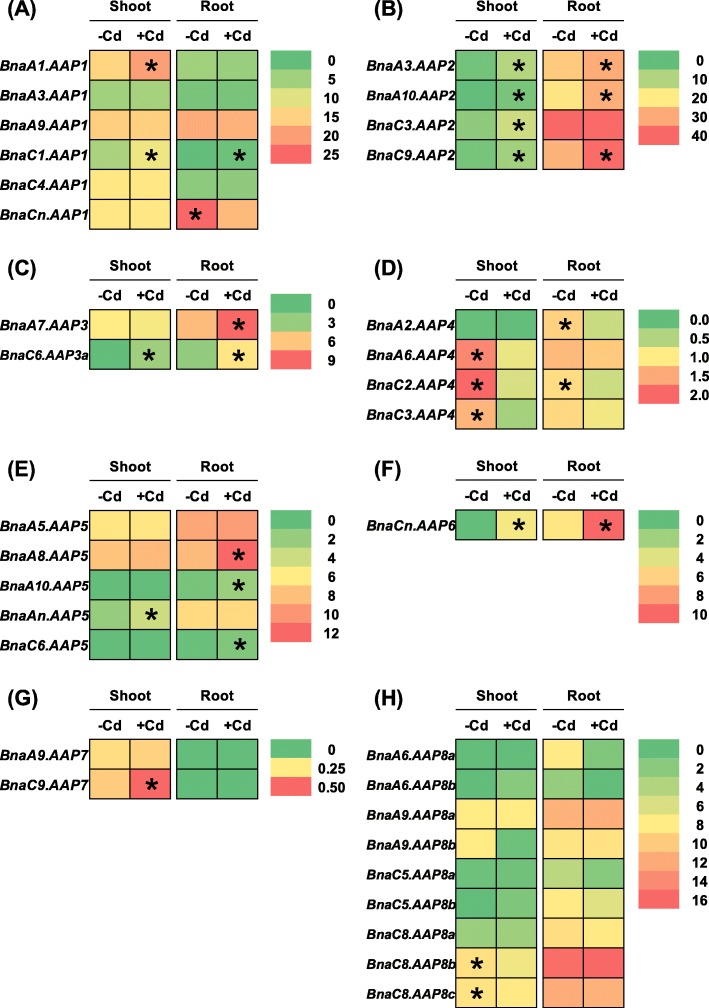
The qRT-PCR-assisted transcriptional characterization of the *amino acid permease* (*AAP*) genes in *Brassica napus* under cadmium (Cd) toxicity. Differential expression of the *BnaAAP1* (**a**), *BnaAAP2* (**b**), *BnaAAP3* (**c**), *BnaAAP4* (**d**), *BnaAAP5* (**e**), *BnaAAP6* (**f**), *BnaAAP7* (**g**) and *BnaAAP8* (**h**) genes under Cd-free (−Cd) and Cd (10 μM CdCl_2_) toxicity. For the transcriptional analysis, the 7-d-old uniform *B. napus* seedlings after seed germination were hydroponically cultivated in a cadmium-free solution for 10 d, and then were transferred to 10 μM CdCl_2_ for 12 h until sampling. The shoots and roots were individually sampled, and each sample includes three independent biological replicates. The significance level of *P* < 0.05 is used as the threshold to identify the differential *BnaAAP* expression under cadmium-free and cadmium treatments. The differentially expressed genes with higher expression between different treatments in the shoots or roots are indicated with asterisks

Cadmium is a non-essential heavy metal that is highly biotoxic for the growth and development of plant species [44]. Rapeseed has great potential for the phytoremediation of cadmium-contaminated soils [45]. Plants exhibit higher concentrations of AAs under cadmium toxicity than under cadmium-free conditions [46]. The increase in AA concentrations under cadmium stresses may be associated with their possible integration into phytochelatins, which serve as metal chelators to alleviate cell damages induced by cadmium toxicity [47, 48]. Under cadmium toxicity, we identified a total of 21 *BnaAAP* DEGs in the shoots and roots (Fig. 11, Additional file 1: Table S2). In the shoots, the expression of the *BnaAAP1*, *BnaAAP2*, *BnaAAP3*, *BnaAAP5*, *BnaAAP6*, and *BnaAAP7* DEGs was significantly elevated under cadmium toxicity (Fig. 11a-c, e-g), which repressed the expression of *BnaAAP4s* and *BnaAAP8s* (Fig. 11d, h). In the roots, the expression of most *BnaAAPs* was significantly upregulated by cadmium toxicity except that the *BnaCn.AAP1* expression was obviously downregulated by cadmium toxicity.

**Fig. 12:**
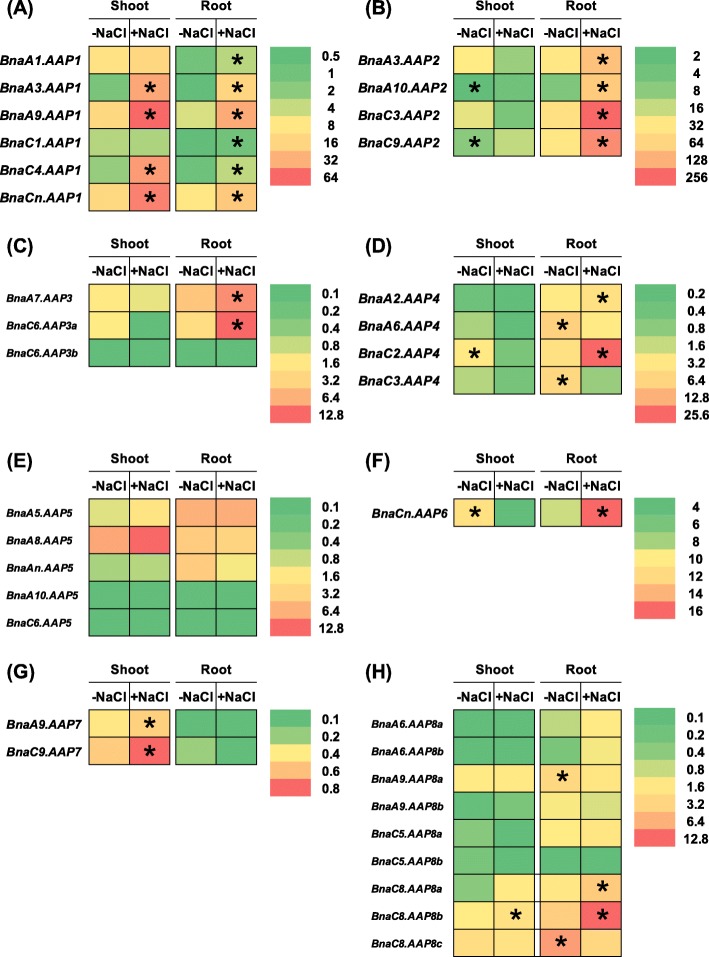
The qRT-PCR-assisted transcriptional characterization of the *amino acid permease* (*AAP*) genes in *Brassica napus* under cadmium (Cd) toxicity. Differential expression of the *BnaAAP1* (**a**), *BnaAAP2* (**b**), *BnaAAP3* (**c**), *BnaAAP4* (**d**), *BnaAAP5* (**e**), *BnaAAP6* (**f**), *BnaAAP7* (**g**) and *BnaAAP8* (**h**) genes under NaCl-free (−NaCl) and salt (+NaCl, 200 mM NaCl) treatments. For the transcriptional analysis, the 7-d-old uniform *B. napus* seedlings after seed germination were hydroponically cultivated in a NaCl-free solution for 10 d, and then were transferred to 200 mM NaCl for 12 h. The shoots and roots were individually sampled, and each sample includes three independent biological replicates. The significance level of *P* < 0.05 is used as the threshold to identify the differential *BnaAAP* expression under cadmium-free and cadmium treatments. The differentially expressed genes with higher expression between different treatments in the shoots or roots are indicated with asterisks

Soil salinity is one of the most important environmental factors that constrain plant growth, and development, and salt stress significantly reduces rapeseed yield [49]. Salt stress induces an obvious increase in AA concentrations [50], and the enhanced *AAP* expression promotes plant salt tolerance [51]. Under salt stress, we identified a total of 23 *AAP* DEGs in the shoots and roots. In the eight *AAP* subgroups, we did not detect differential expression of *BnaAAP5s* (Fig. 12). Most of the DEGs, particularly *BnaAAP1s* and *BnaAAP2*s, showed higher expression levels under salt stress than under the non-salt condition (Fig. 12).

**Fig. 13:**
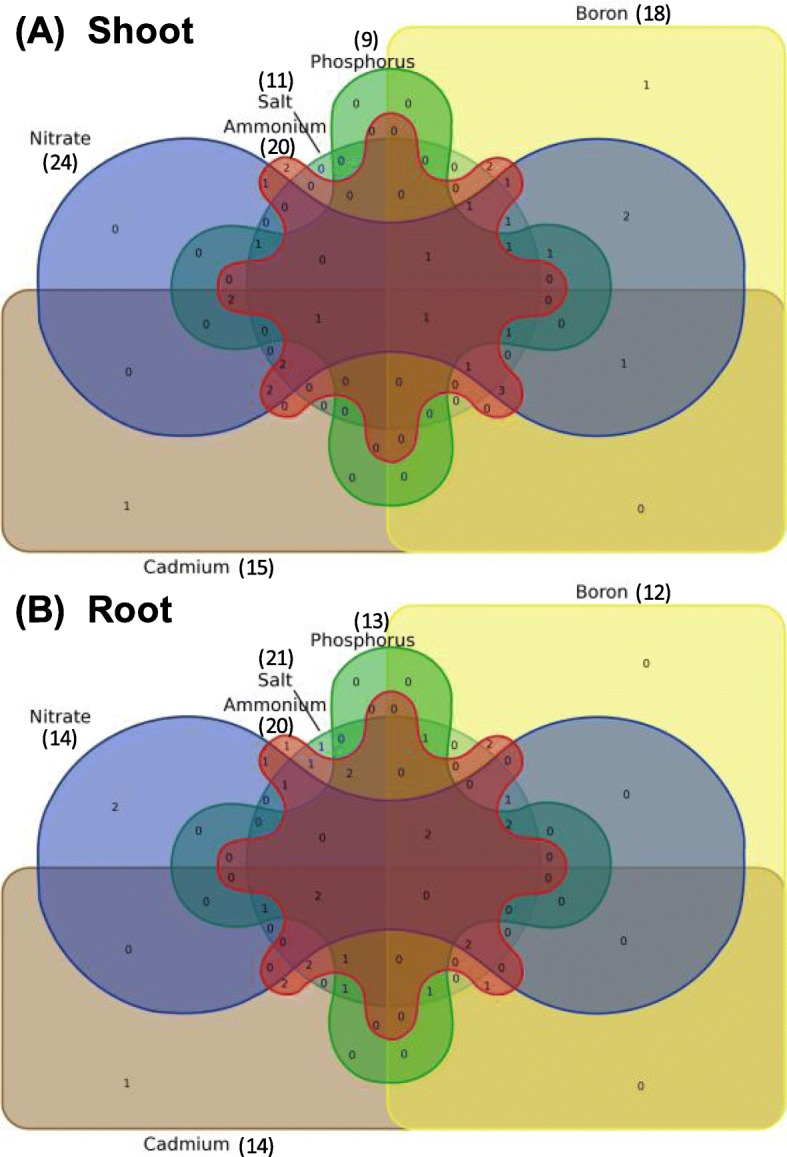
Venn diagram showing the transcriptional responses of the *amino acid permease* (*AAP*) genes in the shoots (**a**) and roots (**b**) of *Brassica napus* under diverse nutrient stresses. The differentially expressed genes between the control and treatments were listed in the brackets

To investigate whether *BnaAAPs* were responsive to diverse nutrient stresses simultaneously, we constructed a Venn diagram with the identified DEGs. As shown in Fig. 13, *BnaC8.AAP8b* was simultaneously regulated by low nitrate, excessive ammonium, limited phosphate, deficient boron, toxic cadmium, and salt stress conditions in the shoots (Fig. 13a). This result indicated that *BnaC8.AAP8b* might play a core role in regulating rapeseed resistance to nutrient stresses through the modulation of AA transport. Although we did not detect any *AAP* homolog simultaneously responsive to these six nutrient stresses in the rapeseed roots, we found several *AAP* homologs, such as *BnaA1.AAP1s* and *BnaA9.AAP1*, simultaneously responsive to three or four nutrient stresses (Fig. 13b).

## Discussion

Previous studies have shown that the *AAP* family members play critical roles in plant growth, organ development, and abiotic and biotic stress responses [4–7]. However, there have been few systematic studies on *AAPs* in *B. napus* so far. In this study, we identified a total of 34 full-length *AAP* homologs representing eight subgroups (*AAP1-AAP8*) in the allotetraploid rapeseed genome (A_n_A_n_C_n_C_n_). We found some homolog number variations of *AAPs* and analyzed their phylogeny relationships in *Brassica* species. In addition, we also analyzed the physio-chemical characteristics, gene/protein structures, conserved AA transport motifs, Darwin’s evolutionary pressure, and CREs of *AAPs* in *B. napus*. Eventually, we delineated the differential expression profile of *BnaAAP*s under nitrate limitation, ammonium excess, phosphate shortage, boron deficiency, cadmium toxicity, and salt stress. The global landscapes of *BnaAAPs* will provide an integrated insight into their family evolution and AAP-mediated AA transport.

### Homolog number variations of *AAPs* in allotetraploid rapeseed indicated their functional divergence

Our previous study has shown that the genes within a family usually exhibit obvious number variations during their evolutionary process [52], which further contributes to gene family division, expansion, and functional divergence [53]. *B. napus*, which is formed by hybridization of the diploid *B. rapa* and *B. oleracea*, undergoes several rounds of whole-genome triplication and duplication compared with Arabidopsis [24]. Therefore, these processes usually causes the formation of multicopy (four to six, or more) gene family in allotetraploid rapeseed [54]. In this study, we found that the homolog number variation events of *AAPs* also occurred in allotetraploid rapeseed (Fig. 1b). In the model Arabidopsis, each *AAP* had a single copy (Fig. 1a), however, the copy number of *BnaAAPs* varied from one (*BnaAAP6*) to nine (*BnaAAP8s*) (Fig. 1b). The result indicated that there might exist the functional divergence of the *BnaAAP* homologs.

### Differential expression profiling of *BnaAAPs* implied their potential involvement in the responses of rapeseed plants to diverse nutrient stresses

Environmental stresses lead to AA accumulation in leaves due to the decrease in AA usage for protein biosynthesis or the increase in AA release from protein degradation [55]. The changes in AA composition in the cells in response to environmental stresses results in the expression alterations of *AATs* [56].

About 75% of the leaf N nutrients in the C_3_ plants are stored in the form of chloroplast proteins [57], which are considered as the main sources for N reallocation within the plant shoots [58]. Under N deficiency, these chloroplast proteins in the old or senescent leaves are degraded into amide-N compounds, particularly AAs, which are reallocated into the newly developing leaves [10]. Therefore, optimal expression of the *AAP* genes will contribute to the efficient transport of AAs, thus favoring the enhancement of crop N limitation adaptation and NUE.

A previous study determined the AA concentrations in all subcellular compartments and characterized the *AAP* family transporters. However, three *AAP* genes were identified and their expression profiling was characterized at different nitrate levels [59]. In another study, 8 *AAP* families (*AAP1-AAP8*) containing 18 *AAP* members were identified in rapeseed, but they does not fully cover the genome-wide *AAP* homologs [60]. Different from our present study, previous studies focused on the *AAP* expression patterns during different growth and developmental stages of *B. napus.*

In this study, we found that most of the *BnaAAP* family genes were upregulated in the shoots or roots under limited nitrate supply (Fig. 7). We speculated that the enhanced expression of *BnaAAPs* might contribute to AA loading into the vascular tissues for long-distance translocation in the roots, and it might facilitate efficient transport of AAs from source leaves to sink organs in the shoots. Ammonium is a major inorganic N source for plants. At low external supplies, ammonium promotes plant growth, whereas at high external supplies it causes toxicity [61]. When plants are exposed to ammonium stresses, excessive ammonium can be involved in the biosynthesis of AAs mainly through the glutamine synthetase/glutamate synthase pathway, and then surplus ammonium or the newly synthesized AAs are translocated from roots to shoots [62]. In this study, we found that the expressions of some *BnaAAPs* were altered in response to ammonium toxicity (Fig. 8), which indicated their potential involvement in the alleviation of excessive ammonium-induced damages in rapeseed plants.

In this study, we found that all of the *BnaAAP* DEGs were upregulated in both the shoots and roots under phosphate deficiency (Fig. 9). It suggested that the phosphate deficiency might facilitate the efficient transport of AA, thus enhancing the adaptation of rapeseed plants to the phosphate-starvation environment. In this study, we found that most of the low boron stress-responsive *BnaAAP*s, such as *BnaAAP1s* and *BnaAAP4–6 s*, were upregulated (Fig. 10). Therefore, it could be concluded that limited boron supply might trigger AA import and translocation in rapeseed plants by enhancing the *AAT* expression. The majority of cadmium-responsive *BnaAAPs*, except *BnaAAP4s* and *BnaAAP8s*, showed higher expression levels under cadmium toxicity than under the cadmium-free condition (Fig. 11). Based on the aforementioned findings, we proposed that the enhanced expression of *BnaAAPs* might contribute to efficient AA transport, and it further facilitated the biosynthesis of cadmium-chelators, therefore enhancing plant cadmium resistance. In addition, we also found that salt stress led to a significant increase in the expression of most *BnaAAPs* in both the shoots and roots (Fig. 12), and might be associated with salt stress inducing an obvious increase in AA concentrations [50], which contributed to enhancing plant salt resistance [51].

Take together, our above results showed that *BnaAAPs* were responsive to diverse nutrient stresses, which implied the essential roles of *BnaAAPs* in the resistance or adaptation of rapeseed plants to stresses.

### Elite *AAPs* could be utilized to enhance crop resistance to biotic and abiotic stresses through genetic engineer

Antisense inhibition of *StAAP1* expression in potato leaves reduces AA content in potato tubers [63]. The over-expression of *PsAAP1* in pea plants contributes to seed AA accumulation and yield enhancement [64]. AAP1-mediated increased proline uptake enhances salt tolerance n Arabidopsis seedlings [51]. Genetic variation in Dip5, an *AAP* member, regulates glyphosate resistance in *Saccharomyces cerevisiae* [65]. Knocking out the Arabidopsis *AAP2* leads to increased N allocation to photosynthetically active source leaves, independently of soil N availability to the plants [15]. The *PsAAP6* function in root nodules affects both shoot and root N supply [6]. All these, the *AAP* family genes play pivotal roles in crop resistance to diverse stresses.

This study characterized global landscapes of *BnaAAPs*, which will provide a comprehensive understanding of this family gene evolution and AAP-mediated AA transport. Our genome-wide identification and molecular characterization of the core *BnaAAPs* may provide elite gene resources and favor the genetic improvement of rapeseed resistance to nutrient stresses, such as nitrogen limitation, ammonium toxicity, phosphate deficiency, boron deficiency, cadmium toxicity and salt stress, through the molecular modulation of *AATs*, particularly *AAPs*.

## Methods

### Retrieval of *AAP* gene sequences

Using the AA sequences of AAPs from *A. thaliana* as sources, we conducted a BLASTp search for the *AAP* homologs in *B. rapa*, *B. oleracea* and *B. napus*. In this study, we retrieved the *AAP* gene sequences using the following databases: The Arabidopsis Information Resource (TAIR10, https://www.arabidopsis.org/) for *A. thaliana*, *Brassica* Database (BRAD) v. 1.1 (http://brassicadb.org/brad/) for *B. rapa* [66], Bol base v. 1.0 (http://119.97.203.210/bolbase/index.html) for *B. oleracea* [67], Genoscope (http://www.genoscope.cns.fr/brassicanapus/) for *B. napus* [24], National Center for Biotechnology Information (NCBI, www.ncbi.nlm.nih.gov), *EnsemblPlants* (http://plants.ensembl.org/index.html), and Phytozome v. 10 (http://phytozome.jgi.doe.gov/pz/portal.html) [68].

### Gene nomenclature of *BnaAAPs*

In this study, based on the nomenclature previously proposed [69–71], we named the *AAP* genes in *Brassica* species following the criterion: genus (one capital letter) + plant species (two lowercase letters) + chromosome (followed by a period) + name of the *AAP* homologs in *A. thaliana*. For example, *BnaA1.AAP1* represents an Arabidopsis *AAP1* homolog on the chromosome A1 of *B. napus*.

### Physical mapping and gene expansion analysis of *BnaAAPs*

We determined the genomic locations of *BnaAAPs* by BLASTn search with the complete nucleotide sequences of *AtAAPs*. Using the genomic annotation, we physically mapped the *AtAAPs* and *BnaAAPs* onto the chromosomes using the MapGene2Chromosome v2.1 (http://mg2c.iask.in/mg2c_v2.1/). In this study, we defined the tandem duplicated genes as an array of two or more *AAP* genes within a 100-kb genomic region.

### Multiple sequence alignment and phylogeny analysis

We aligned the full-length sequences of the AAP proteins of Arabidopsis and *B. napus* using ClustalW [72] within MEGA (Molecular Evolutionary Genetics Analysis) v. 6.06 (http://www.megasoftware.net/) [73]. After these alignments, we constructed the phylogenetic trees with the neighbor-joining (NJ) method [74]. We set the Poisson correction, pairwise deletion, and bootstrapping (1000 replicates; random seeds) as the required parameters.

### Analysis of evolutionary selection pressure and functional divergence of *BnaAAPs*

To determine positive or negative (purifying) selection pressure on *BnaAAPs*, we calculated the values of Ks, Ka, and Ka/Ks. First, we performed pairwise alignment of the *BnaAAP-AtAAP* CDSs using Clustal Omega (http://www.clustal.org/omega/) [75]. Then, we submitted the readout to the KaKs_Calculator (https://sourceforge.net/projects/kakscalculator2/) software [76] for the calculation of the Ka, Ks, and Ka/Ks with the yn00 method [77]. According to the Darwin’s evolution theory, it is proposed that Ka/Ks > 1.0 means positive selection, while Ka/Ks < 1.0 indicates purifying selection, and Ka/Ks = 1.0 denotes neutral selection. Further, we calculated the divergence time of *BnaAAPs* from their progenitors by the following formula: $\mathsf{T}=\mathsf{Ks}/\mathsf{2}\lambda, \lambda =\mathsf{1.5}\times {\mathsf{10}}^{-\mathsf{8}}\mathsf{forBrassicaceaespecies}$ [78].

### Molecular characterization of BnaAAPs

To reveal the molecular characteristics of BnaAAPs, we used the ExPASy ProtoParam (http://www.expasy.org/tools/protparam.html) [79] program to determine the AA number and composition, MW, pI, GRAVY, and IIs. Values of II > 40.0 suggest that the proteins are unstable [35]. We used the online WoLF PSORT (http://www.genscript.com/wolf-psort.html) [80] program to predict the subcellular localization. To characterize the transmembrane helices of AtAAPs and BnaAAPs, we submitted their AA sequences to the TMHMM v. 2.0 (http://www.cbs.dtu.dk/services/TMHMM/) program.

We employed the online SignalP v. 4.1 (http://www.cbs.dtu.dk/services/SignalP/) [81] to predict the presence and location of signal peptide cleavage sites in the AA sequences of BnaAAPs. To determine the recombinant protein solubility, we used the RPSP v. 2009 (http://biotech.ou.edu) program, in which the AAP proteins are assumed to be overexpressed in *E. coli* [82].

We used the STRING (Search Tool for Recurring Instances of Neighboring Genes) v 11.0 (https://string-db.org) [83] web-server to retrieve and display the repeatedly occurring association networks, including direct (physical) and indirect (function) association, of the AAP proteins in *A. thaliana* and *B. napus*.

### Conserved motif/domain identification of BnaAAPs

We used the online InterProScan5 (http://www.ebi.ac.uk/interpro/search/sequence-search) and the conserved domain database (CDD) (http://www.ncbi.nlm.nih.gov/Structure/bwrpsb/bwrpsb.cgi) to determine the presence of the Aa_trans domain (PAFM01490) of the AAP proteins.

To further examine the structural divergence of the AAP proteins in *A. thaliana* and *Brassica* crops, we submitted the protein sequences to the online MEME (Multiple Expectation maximization for Motif Elicitation) v. 4.12.0 (http://meme-suite.org/tools/meme) [84] for the characterization of conserved motifs/domains. We used all the default parameters except the following two parameters: the optimum motif width was set as 6–50 bp and the maximum number of motifs was set as 10. The conserved motif sequences were presented by the online Weblogo (https://weblogo.berkeley.edu/logo.cgi) [85].

### Elucidation of exon-intron structure and putative CREs in promoter regions of *BnaAAPs*

Full-length genomic DNA (gDNA) and CDS sequences were collected from the annotated genomes of *A. thaliana* and *B. napus*, and they were used to predict the exon-intron structure of *AAP* genes. For each *AAP* gene, a 2.0-kb genomic sequence upstream from the start codon (ATG) was downloaded from the TAIR (https://www.arabidopsis.org/) website and *B. napus* Genome Browser (http://www.genoscope.cns.fr/brassicanapus/) [24]. Subsequently, we submitted these sequences to the PLACE v. 30.0 (http://www.dna.affrc.go.jp/PLACE/) program [86] to identify putative CREs.

### Transcriptional analysis of *BnaAAPs* under diverse nutrient stresses

In this study, the expression patterns of *AtAAPs* were obtained from the TAIR eFP Browser [87]. To further characterize the transcriptional responses of *BnaAAPs* under diverse nutrient stresses, we transplanted the 7-d-old uniform *B. napus* seedlings (Zhongshuang 11) after seed germination into black plastic containers with 10 L Hoagland nutrient solution. The basic nutrition solution contained 1.0 mM KH_2_PO_4_, 5.0 mM KNO_3_, 5.0 mM Ca(NO_3_)_2_·4H_2_O, 2.0 mM MgSO_4_·7H_2_O, 0.050 mM EDTA-Fe, 9.0 μM MnCl_2_·4H_2_O, 0.80 μM ZnSO_4_·7H_2_O, 0.30 μM CuSO_4_·5H_2_O, 0.10 μM Na_2_MoO_4_·2H_2_O, and 46 μM H_3_BO_3_. The rapeseed seedlings were cultivated in an illuminated chamber following the growth regimes: light intensity of 300–320 μmol m^− 2^ s^− 1^, temperature of 25 °C daytime/22 °C night, light period of 16 h photoperiod/ 8 h dark, and relative humidity of 70%.

For the nitrate (NO_3_^−^) depletion treatment, the 7-d-old uniform *B. napus* seedlings were hydroponically cultivated under high (6.0 mM) nitrate for 10 d, and then were grown under low (0.30 mM) nitrate for 3 d until sampling. For the ammonium (NH_4_^+^) toxicity treatment, the 7-d-old uniform *B. napus* seedlings after seed germination were hydroponically cultivated under high nitrate (6.0 mM) for 10 d, and then were grown under N-free condition for 3 d. Finally, the plants were grown under excess ammonium (9.0 Mm NH_4_^+^) for 6 h until sampling. For the inorganic phosphate (Pi) starvation treatment, the 7-d-old uniform *B. napus* seedlings after seed germination were first hydroponically grown under 250 μM phosphate (KH_2_PO_4_) for 10 d, and then were grown under 5 μM phosphate for 3 d until sampling. For the boron (B) deficiency treatment, the 7-d-old uniform *B. napus* seedlings after seed germination were first hydroponically grown under 10 μM H_3_BO_3_ for 10 d, and then were transferred to 0.25 μM H_3_BO_3_ for 3 d until sampling. For the cadmium (Cd) toxicity treatment, the 7-d-old uniform *B. napus* seedlings after seed germination were hydroponically cultivated in a Cd-free solution for 10 d, and then were transferred to 10 μM CdCl_2_ for 12 h until sampling. For the salt stress treatment, the 7-d-old uniform *B. napus* seedlings after seed germination were hydroponically cultivated in a NaCl-free solution for 10 d, and then were transferred to 200 mM NaCl for 1 d until sampling. The shoots and roots were individually harvested and immediately stored at − 80 °C until RNA isolation. Each sample contained three independent biological replicates for the transcriptional analyses of *BnaAAPs* under diverse nutrient stresses.

### Quantitative reverse-transcription PCR assays

The quantitative reverse-transcription polymerase chain reaction (qRT-PCR) assays were used to determine the relative expression of *BnaAAP*s. After removing genomic DNA from the RNA samples with RNase-free DNase I, complementary DNA (cDNA) synthesis was performed using the PrimeScript™ RT reagent Kit with gDNA Eraser (Perfect Real Time) (TaKaRa, Shiga, Japan) with total RNA as the templates. We performed the quantitative analysis of relative gene expression by using the SYBR® *Premix Ex Taq*™ II (Tli RNaseH Plus) (TaKaRa, Shiga, Japan) kit in an Applied Biosystems StepOne™ Plus Real-time PCR System (Thermo Fisher Scientific, Waltham, MA, USA). The thermal recycle regimes were as follows: 95 °C for 3 min, followed by 40 cycles of 95 °C for 10 s, then 60 °C for 30 s [37]. We also conducted a melting curve analysis to ensure the primer specificity of target genes: 95 °C for 15 s, 60 °C for 1 min, and 60 °C–95 °C for 15 s (+ 0.3 °C per cycle). The public gene *BnaEF1-α* (forward: GCCTGGTATGGTTGTGACCT; reverse: GAAGTTAGCAGCACCCTTGG) [88] was used as internal references, and the gene expression abundances that were calculated with the slightly modified 2^-ΔΔC^_*T*_ method [89], and the results were showed by heat maps. To ensure the result consistency, we also used a second reference gene *BnaGDI1* (forward: GAGTCCCTTGCTCGTTTCC; reverse: TGGCAGTCTCTCCCTCAGAT) [90] to confirm the gene expression consistency. The gene-specific primers for the qRT-PCR assays were listed in the additional file: Table S3.

### Statistical analysis

Statistical analysis was performed using the Student’s *t*-test, followed by the Tukey’s honestly significant difference (HSD) tests, with the Statistical Productions and Service Solutions (SPSS) 17.0 toolkit. Considering that the qRT-PCR data are normally distributed without log-transformation, we determined the significant difference (*P* < 0.05) in the expression of the target genes using the raw data.

## Supplementary information

**Additional file 1: Table S1.** Molecular characterization of the amino acid permease (AAP) proteins in *Brassica napus***. Table S2.** Differential expression of each *amino acid permease* (*AAP*) gene under diverse nutrient stresses. **Table S3.** Raw expression data for the *amino acid permease (AAP)* family genes under diverse nutrient stresses. **Figure S1.** Rooted phylogeny analysis of the *AAP* genes in allotetraploid rapeseed. **Figure S2.** Trans-membrane characterization of the amino acid permease (AAP) proteins in *Arabidopsis thaliana*. The TMHMM (http://www.cbs.dtu.dk/services/TMHMM/) tool was used to predict the transmembrane topology of the AtAAP proteins. **Figure S3.** Characterization of signal peptides of the amino acid permease (AAP) proteins in *Arabidopsis thaliana*. The SignalP (http://www.cbs.dtu.dk/services/SignalP/) 4.0 server was used to predict the presence and location of signal peptide cleavage sites in the amino acid sequences of the AtAAP proteins. **Figure S4.** Tissue-specific expression patterns of the *amino acid permease* (*AAP*) genes in *Arabidopsis thaliana*. (A-H) Relative expression abundances of *AtAAP1* (A), *AtAAP2* (B), *AtAAP3* (C), *AtAAP4* (D), *AtAAP5* (E), *AtAAP6* (F), *AtAAP7* (G) and *AtAAP8* (H) in various tissues. The red and yellow color indicates relative high and low expression levels of *AtAAPs*.

## Data Availability

All the data and materials that are required to reproduce these findings can be shared by contacting the corresponding author, Dr. Ying-peng Hua (yingpenghua@zzu.edu.cn).

## Abbreviations

AA: Amino acid
AAP: Amino acid permease
AAT: Amino acid transporter
*At*: *Arabidopsis thaliana*
B: Boron
*Bna*: *Brasssica napus*
*Bol*: *Brassica oleracea*
*Bra*: *Brassica rapa*
BRAD: *Brassica* Database
Cd: Cadmium
CNV: Copy number variation
CRE: *Cis*-acting regulatory element
DEGs: Differentially expressed genes
DGE: Differential gene expression
gDNA: Genomic DNA
N: Nitrogen
NCBI: National Center for Biotechnology Information
NH_4_^+^: Ammonium
NO_3_^−^: Nitrate
NUE: Nitrogen use efficiency
Pi: Phosphate
qRT-PCR: Quantitative reverse-transcription polymerase chain reaction;
TAIR: The Arabidopsis Information Resource;
TF: Transcription factor
