# Supplementary material for: Genome-wide identification of the amino acid permease genes and molecular characterization of their transcriptional responses to various nutrient stresses in allotetraploid rapeseed
Source: BMC Plant Biol. 2020 Apr 8;20:151. doi: 10.1186/s12870-020-02367-7 (PMC7140331; doi:10.1186/s12870-020-02367-7)
Supplement: Supplementary file 1 — Additional file 1: Table S1. Molecular characterization of the amino acid permease (AAP) proteins in Brassica napus. Table S2. Differential expression of each amino acid permease (AAP) gene under diverse nutrient stresses. Table S3. Raw expression data for the amino acid permease (AAP) family genes under diverse nutrient stresses. Figure S1. Rooted phylogeny analysis of the AAP genes in allotetraploid rapeseed. Figure S2. Trans-membrane characterization of the amino acid permease (AAP) proteins in Arabidopsis thaliana. The TMHMM (http://www.cbs.dtu.dk/services/TMHMM/) tool was used to predict the transmembrane topology of the AtAAP proteins. Figure S3. Characterization of signal peptides of the amino acid permease (AAP) proteins in Arabidopsis thaliana. The SignalP (http://www.cbs.dtu.dk/services/SignalP/) 4.0 server was used to predict the presence and location of signal peptide cleavage sites in the amino acid sequences of the AtAAP proteins. Figure S4. Tissue-specific expression patterns of the amino acid permease (AAP) genes in Arabidopsis thaliana. (A-H) Relative expression abundances of AtAAP1 (A), AtAAP2 (B), AtAAP3 (C), AtAAP4 (D), AtAAP5 (E), AtAAP6 (F), AtAAP7 (G) and AtAAP8 (H) in various tissues. The red and yellow color indicates relative high and low expression levels of AtAAPs. [file 12870_2020_2367_MOESM1_ESM.zip › Additional file 1_ Table S1-2; Figure S1-4.docx]

**Additional file 1**


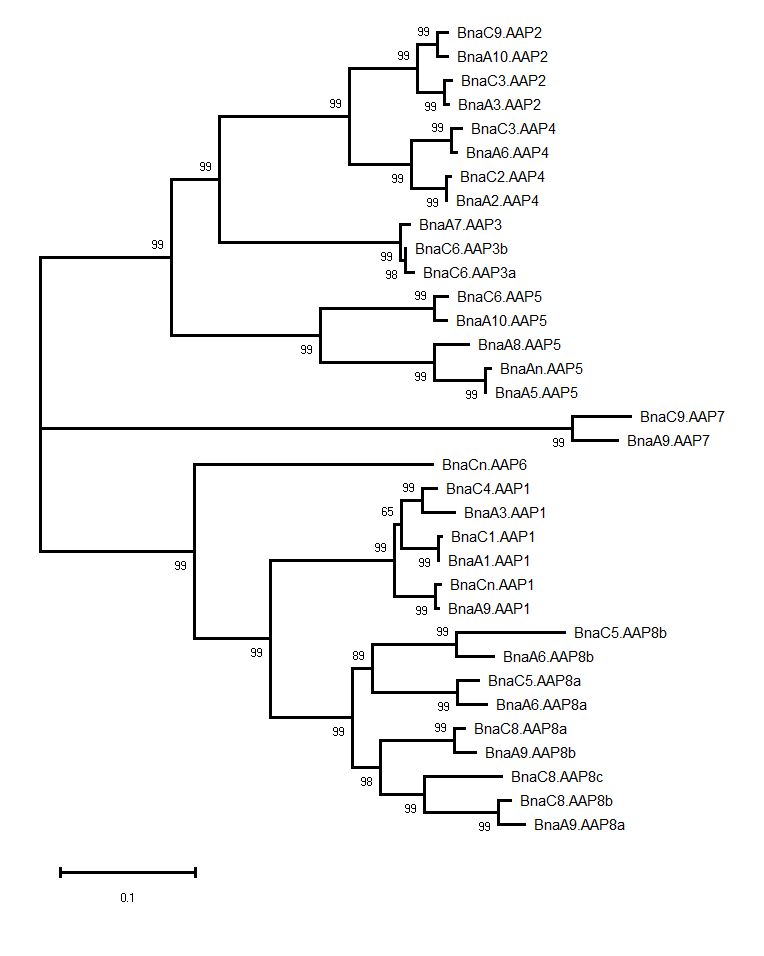


**Figure S1** Rooted phylogeny analysis of the *AAP* genes in allotetraploid rapeseed. The evolutionary history was inferred using the Neighbor-Joining method. The optimal tree with the sum of branch length = 2.61 is shown. The confidence probability (multiplied by 100) that the interior branch length is greater than 0, as estimated using the bootstrap test (500 replicates is shown next to the branches [2, 3]. The tree is drawn to scale, with branch lengths in the same units as those of the evolutionary distances used to infer the phylogenetic tree. The evolutionary distances were computed using the Poisson correction method and are in the units of the number of amino acid substitutions per site. This analysis involved 34 amino acid sequences. All ambiguous positions were removed for each sequence pair (pairwise deletion option). There were a total of 1096 positions in the final dataset.

**Figure S2** Trans-membrane characterization of the amino acid permease (AAP) proteins in *Arabidopsis thaliana*. The TMHMM (http://www.cbs.dtu.dk/services/TMHMM/) tool was used to predict the transmembrane topology of the AtAAP proteins.

**Figure S3** Characterization of signal peptides of the amino acid permease (AAP) proteins in *Arabidopsis thaliana*. The SignalP (http://www.cbs.dtu.dk/services/SignalP/) 4.0 server was used to predict the presence and location of signal peptide cleavage sites in amino acid sequences of the AtAAP proteins.

**Figure** **S4** Tissue-specific expression patterns of the *amino acid permease* (*AAP*) genes in *Arabidopsis thaliana*. (A-H) Relative expression abundances of *AtAAP1* (A), *AtAAP2* (B), *AtAAP3* (C), *AtAAP4* (D), *AtAAP5* (E), *AtAAP6* (F), *AtAAP7* (G) and *AtAAP8* (H) in various tissues. The red and yellow color indicates relative high and low expression levels of *AtAAPs*.

**Table S1** Molecular characterization of the amino acid permease (AAP) proteins in *Brassica napus*

| Gene name | | MW | pI | II | GRAVY |
| --- | --- | --- | --- | --- | --- |
| *AAP1* | *BnaA1.AAP1* | 55.9 | 8.96 | 33.9 | 0.422 |
|  | *BnaA3.AAP1* | 59.7 | 6.19 | 35.5 | 0.409 |
|  | *BnaA9.AAP1* | 52.5 | 9.17 | 32.9 | 0.375 |
|  | *BnaC1.AAP1* | 55.9 | 8.96 | 36.1 | 0.400 |
|  | *BnaC4.AAP1* | 53.1 | 8.99 | 34.8 | 0.420 |
|  | *BnaCn.AAP1* | 52.5 | 9.17 | 33.5 | 0.420 |
| *AAP2* | *BnaA3.AAP2* | 53.4 | 8.95 | 31.9 | 0.437 |
|  | *BnaA10.AAP2* | 53.1 | 9.08 | 35.4 | 0.482 |
|  | *BnaC3.AAP2* | 53.5 | 8.95 | 33.8 | 0.499 |
|  | *BnaC9.AAP2* | 53.2 | 9.05 | 38.1 | 0.506 |
| *AAP3* | *BnaA7.AAP3* | 52.1 | 8.94 | 38.3 | 0.474 |
|  | *BnaC6.AAP3a* | 52.2 | 9.01 | 37.2 | 0.479 |
|  | *BnaC6.AAP3b* | 52.2 | 9.01 | 37.0 | 0.465 |
| *AAP4* | *BnaA2.AAP4* | 51.2 | 9.17 | 37.8 | 0.491 |
|  | *BnaA6.AAP4* | 51.3 | 9.07 | 36.7 | 0.538 |
|  | *BnaC2.AAP4* | 51.2 | 9.16 | 39.9 | 0.488 |
|  | *BnaC3.AAP4* | 51.4 | 9.19 | 35.4 | 0.539 |
| *AAP5* | *BnaA5.AAP5* | 53.3 | 8.94 | 33.8 | 0.485 |
|  | *BnaA8.AAP5* | 52.8 | 8.86 | 34.9 | 0.431 |
|  | *BnaA10.AAP5* | 51.7 | 9.04 | 35.6 | 0.426 |
|  | *BnaAn.AAP5* | 45.8 | 9.03 | 39.1 | 0.529 |
|  | *BnaC6.AAP5* | 51.8 | 8.89 | 39.1 | 0.529 |
| *AAP6* | *BnaCn.AAP6* | 52.5 | 9.18 | 34.3 | 0.372 |
| *AAP7* | *BnaA9.AAP7* | 51.7 | 9.21 | 31.6 | 0.503 |
|  | *BnaC9.AAP7* | 38.4 | 8.73 | 38.4 | 0.559 |
| *AAP8* | *BnaA6.AAP8a* | 50.5 | 9.17 | 36.2 | 0.477 |
|  | *BnaA6.AAP8b* | 51.0 | 9.28 | 29.4 | 0.520 |
|  | *BnaA9.AAP8a* | 55.3 | 9.05 | 27.8 | 0.508 |
|  | *BnaA9.AAP8b* | 52.4 | 8.95 | 32.2 | 0.526 |
|  | *BnaC5.AAP8a* | 52.4 | 8.95 | 31.0 | 0.514 |
|  | *BnaC5.AAP8b* | 51.1 | 8.98 | 31.5 | 0.566 |
|  | *BnaC8.AAP8a* | 52.4 | 9.02 | 29.3 | 0.360 |
|  | *BnaC8.AAP8b* | 54.7 | 8.93 | 30.2 | 0.526 |
|  | *BnaC8.AAP8c* | 52.2 | 9.10 | 31.1 | 0.535 |

**Table S2** Differential expression of each *amino acid permease* gene under diverse nutrient stresses

| Gene name | Nitrate | | Ammonium | | | Phosphate | | | Boron | | | Cadmium | | | NaCl | |
| --- | --- | --- | --- | --- | --- | --- | --- | --- | --- | --- | --- | --- | --- | --- | --- | --- |
|  | Shoot | Root | | Shoot | Root | | Shoot | Root | | Shoot | Root | | Shoot | Root | Shoot | Root |
| *BnaA1.AAP1* | Up | Up | | Down | Down | | ns | Up | | ns | Up | | Up | ns | ns | Up |
| *BnaA3.AAP1* | Up | Up | | ns | Up | | Un | Un | | Up | ns | | ns | ns | Up | Up |
| *BnaA9.AAP1* | Up | Up | | ns | Down | | Up | Up | | Up | Up | | ns | ns | Up | Up |
| *BnaC1.AAP1* | Up | Up | | Down | Down | | Un | Un | | Up | Up | | Up | Up | ns | Up |
| *BnaC4.AAP1* | Up | Up | | ns | ns | | Up | ns | | ns | Up | | ns | ns | Up | Up |
| *BnaCn.AAP1* | Up | Up | | Down | Down | | Un | Un | | Up | Up | | ns | Down | Up | Up |
| *BnaA3.AAP2* | Up | ns | | Up | Up | | Up | ns | | ns | ns | | Up | Up | ns | Up |
| *BnaA10.AAP2* | Up | ns | | Up | Up | | ns | Up | | ns | ns | | Up | Up | Down | Up |
| *BnaC3.AAP2* | Up | ns | | Up | Up | | ns | Up | | ns | ns | | Up | ns | ns | Up |
| *BnaC9.AAP2* | Up | ns | | Up | Up | | Up | ns | | ns | ns | | Up | Up | Down | Up |
| *BnaA7.AAP3* | Up | Down | | ns | Down | | ns | Down | | Down | ns | | ns | Up | ns | Up |
| *BnaC6.AAP3a* | Up | Up | | ns | ns | | ns | Down | | Down | ns | | Up | Up | ns | Up |
| *BnaC6.AAP3b* | Un | Un | | Un | Un | | Un | Un | | Down | ns | | Un | ns | ns | ns |
| *BnaA2.AAP4* | Up | ns | | ns | ns | | Up | Up | | Up | ns | | ns | Down | ns | Up |
| *BnaA6.AAP4* | ns | ns | | ns | Up | | ns | Up | | ns | ns | | Down | ns | ns | Down |
| *BnaC2.AAP4* | Up | ns | | ns | ns | | Up | Up | | Up | Down | | Down | Down | Down | Up |
| *BnaC3.AAP4* | Up | Up | | Up | ns | | ns | Up | | Up | Up | | Down | ns | ns | Down |
| *BnaA5.AAP5* | Up | ns | | Up | Up | | ns | ns | | Up | Up | | ns | ns | ns | ns |
| *BnaA8.AAP5* | Up | ns | | Up | Up | | ns | ns | | Up | Up | | ns | Up | ns | ns |
| *BnaA10.AAP5* | Up | ns | | ns | Up | | ns | ns | | ns | ns | | ns | Up | ns | ns |
| *BnaAn.AAP5* | ns | ns | | Up | Up | | Un | ns | | ns | ns | | Up | ns | ns | ns |
| *BnaC6.AAP5* | ns | ns | | Up | Up | | ns | ns | | ns | Up | | ns | Up | ns | ns |
| *BnaCn.AAP6* | Up | Down | | Up | Up | | ns | Up | | Up | ns | | Up | Up | Down | Up |
| *BnaA9.AAP7* | Up | ns | | Up | ns | | Up | ns | | Up | ns | | ns | ns | Up | ns |
| *BnaC9.AAP7* | ns | ns | | Up | ns | | ns | ns | | ns | ns | | Up | ns | Up | ns |
| *BnaA6.AAP8a* | ns | Up | | ns | Up | | Un | Un | | Un | Un | | ns | ns | ns | ns |
| *BnaA6.AAP8b* | ns | Up | | Un | Un | | Un | Un | | Un | Un | | ns | ns | ns | ns |
| *BnaA9.AAP8a* | ns | ns | | Down | Down | | Un | Un | | Up | ns | | ns | ns | ns | Down |
| *BnaA9.AAP8b* | ns | Up | | Down | ns | | Un | Un | | Up | ns | | ns | ns | ns | ns |
| *BnaC5.AAP8a* | ns | ns | | ns | ns | | ns | ns | | Un | Un | | ns | ns | ns | ns |
| *BnaC5.AAP8b* | ns | ns | | ns | Up | | Un | Un | | Un | Un | | ns | ns | ns | ns |
| *BnaC8.AAP8a* | ns | Up | | ns | ns | | ns | Up | | Up | Down | | ns | ns | ns | Up |
| *BnaC8.AAP8b* | Up | ns | | Down | ns | | Up | Up | | Up | Down | | Down | ns | Up | Up |
| *BnaC8.AAP8c* | Up | ns | | Down | ns | | Up | ns | | ns | ns | | Down | ns | ns | Down |

**Table S3** The gene-specific primers for the qRT-PCR assays in this study

| Gene name | | Forward sequence (5'-3') | Reverse sequence (5'-3') |
| --- | --- | --- | --- |
| *AAP1* | *BnaA1.AAP1* | GGTGCTTATCAGGTGTTTGCC | GAAAGGGAATATCATAGCTACCACC |
|  | *BnaA3.AAP1* | CGAGTCCAATCCTACAAAGAACA | GCACGGTAGCAATCAGCGAG |
|  | *BnaA9.AAP1* | CGCATATTATCACGGCGGTA | TGTAGTTTCGTTTTCCGGTGAG |
|  | *BnaC1.AAP1* | AGTATCAACCACCACTTTCTTCTACATA | AACAAACTGGAAGATGGGCTGT |
|  | *BnaC4.AAP1* | AAATCTATGAAAAGAGCAAGCCTTC | ACCTGATAAGCACCGATAAGGTG |
|  | *BnaCn.AAP1* | GCACCATGCAGATTGTACAATATCA | TGGCTAGGCCAATCCCAATAC |
| *AAP2* | *BnaA3.AAP2* | GGTACTGTTTGGACCGCAAGT | AGCCCACATATCTTGAACTTGAAT |
|  | *BnaA10.AAP2* | ATATCATTACAGCAGTTATCGGATCC | TCAGCCCACAGATCTTGAACTTA |
|  | *BnaC3.AAP2* | GGTTCAGGCGTTCTCTCGC | TGAACCCACCGAGAATGGAC |
|  | *BnaC9.AAP2* | GGCGTTCTCTCGTTGGCG | CCACCGAGAATGGATCGG |
| *AAP3* | *BnaA7.AAP3* | AAAACCAGCAAGAAGCTTTCG | GACGATGGAGAAGAGCAACATC |
|  | *BnaC6.AAP3a* | CCTTACATGATAGCTTTTGGTCTG | ACCAACGAGGTGAACCACAAT |
|  | *BnaC6.AAP3b* | CCTTACATGATAGCTTTTGGTCTG | ACCAACGAGGAGAACCACAAC |
| *AAP4* | *BnaA2.AAP4* | CGCAAGTGCGCATATTATAACA | CCAAAAAGATTCAAGAACTGAATCAG |
|  | *BnaA6.AAP4* | GCTGGCATGGGCCATAGC | GTACCGAAAAGATTCAAGAACTGC |
|  | *BnaC2.AAP4* | CTCTAGCATGGGCCATAGGC | TGGAAGCAGTTCGATCTCTTAATT |
|  | *BnaC3.AAP4* | ACATGGATGCTGTCCAATCAATT | CGGATTGCTTGACATGTGACATA |
| *AAP5* | *BnaA5.AAP5* | GCACATATCATTACAGCAGTTATTGGT | ACCTTAATGCCACCGAGGTTC |
|  | *BnaA8.AAP5* | TCGACGAAGATGGCCGTGT | GAGAATAGAAGTGTAATAAGTGACGAGG |
|  | *BnaA10.AAP5* | GATGATGGCCGTCCCAAA | AATAGCCCATGCCAGAGACAGT |
|  | *BnaAn.AAP5* | AGTCATGTCTTTTGGTTACTCCACC | CATGTAGAAAAGCGTTGTCACTACT |
|  | *BnaC6.AAP5* | CCTCGAAGCAGCTCCGAG | CGGTGACAGGGTCCCCA |
| *AAP6* | *BnaCn.AAP6* | TGACGATGATGGACGCCAG | GGCAAGCATGGTTGATGTAAAG |
| *AAP7* | *BnaA9.AAP7* | AACGAGTCTCCAGTTTTGAGATCT | GGCGGCAGGACCACCT |
|  | *BnaA9.AAP7* | AACGAGTCTCCAGTTTTGAGATCA | GCGGAGGGGACCGTTTA |
| *AAP8* | *BnaA6.AAP8a* | AAGTGGAAAGGTTGGTAAGACGA | CATGACTTTGTTTTCTGGTGGGT |
|  | *BnaA6.AAP8b* | TCCGAGCTTACCTTGGTGGTAA | GGCATGGAACATTTCGCC |
|  | *BnaA9.AAP8a* | TGTTTTCATGACAAGGGACATGA | ATTTTGTCAGACGCAGTTACGTCT |
|  | *BnaA9.AAP8b* | CACGGTGGTTGGAGTGGAT | TGTAGAAAGCAGTTGTAGTTGAGACTG |
